# Identifying a Serum Exosomal-Associated lncRNA/circRNA-miRNA-mRNA Network in Coronary Heart Disease

**DOI:** 10.1155/2021/6682183

**Published:** 2021-06-23

**Authors:** Jia Mao, Yufei Zhou, Licheng Lu, Ping Zhang, Runhua Ren, Yaqiong Wang, Jing Wang

**Affiliations:** ^1^Emergency Department, The Affiliated Wuxi No. 2 People's Hospital of Nanjing Medical University, Wuxi 214000, Jiangsu, China; ^2^Department of Cardiology, The First Affiliated Hospital of Nanjing Medical University, Nanjing 210029, Jiangsu, China; ^3^Department of Cardiology, Kunshan Hospital of Traditional Chinese Medicine, Kunshan 215300, Jiangsu, China; ^4^Department of Geriatric Medicine, The Affiliated Jiangning Hospital with Nanjing Medical University, Nanjing 211100, Jiangsu, China

## Abstract

**Background:**

Accumulating evidence supports the importance of noncoding RNAs and exosomes in coronary heart disease (CHD). However, exosomal-associated competing endogenous RNA- (ceRNA-) mediated regulatory mechanisms in CHD are largely unexplored. The present study aimed to explore exosomal-associated ceRNA networks in CHD.

**Methods:**

Data from 6 CHD patients and 32 normal controls were downloaded from the ExoRBase database. CHD and normal controls were compared by screening differentially expressed mRNAs (DEMs), lncRNAs (DELs), and circRNAs (DECs) in serum exosomes. MicroRNAs (miRNAs) targeting DEMs were predicted using the Targetscan and miRanda databases, and miRNAs targeted by DELs and DECs were predicted using the miRcode and starBase databases, respectively. The biological functions and related signaling pathways of DEMs were analyzed using the David and KOBAS databases. Subsequently, a protein-protein interaction (PPI) network was established to screen out on which hub genes enrichment analyses should be performed, and a ceRNA network (lncRNA/circRNA-miRNA-mRNA) was constructed to elucidate ceRNA axes in CHD.

**Results:**

A total of 312 DEMs, 43 DELs, and 85 DECs were identified between CHD patients and normal controls. Functional enrichment analysis showed that DEMs were significantly enriched in “chromatin silencing at rDNA,” “telomere organization,” and “negative regulation of gene expression, epigenetic.” PPI network analysis showed that 25 hub DEMs were closely related to CHD, of which ubiquitin C (UBC) was the most important. Hub genes were mainly enriched in “cellular protein metabolic process” functions. The exosomal-associated ceRNA regulatory network incorporated 48 DEMs, 73 predicted miRNAs, 10 DELs, and 15 DECs. The LncRNA/circRNA-miRNA-mRNA interaction axes (RPL7AP11/hsa-miR-17-5p/UBC and RPL7AP11/hsa-miR-20b-5p/UBC) were obtained from the network.

**Conclusions:**

Our findings provide a novel perspective on the potential role of exosomal-associated ceRNA network regulation of the pathogenesis of CHD.

## 1. Introduction

Coronary heart disease (CHD) is a complex biological process accompanied by a wide range of transcriptional changes, but the mechanism of CHD is still complex and unclear [1].

Noncoding RNAs mainly consist of microRNAs (miRNAs/miRs), long noncoding RNAs (lncRNAs), and circular RNAs (circRNAs). MiRNAs are a class of small noncoding RNAs that can block protein translation or induce degradation by targeting specific regions of messenger RNA (mRNA) [2]. LncRNAs have more diverse functions as epigenetic regulators, molecular scaffolds, and decoys [3]. CircRNAs can function as templates for viroid and viral replication, as intermediates in RNA processing, as regulators of transcription, as small nucleolar RNAs, and as miRNA sponges [4]. With the development of sequencing technology and bioinformatics, it has been found that noncoding RNAs are involved in the pathophysiology of cardiovascular diseases [5]. Ahmadi et al. [6] demonstrated that miR-342-5p could be a biomarker for diagnosis of CHD associated with inflammatory cytokines. Wang et al. [7] revealed that BRAF-activated lncRNA is associated with CHD. Moreover, circRNAs or lncRNAs have been found to interact with miRNAs as competing endogenous RNAs (ceRNAs) to regulate target mRNA activity in CHD. For example, *YOD1* deubiquitinase might be a novel target for diagnosing CHD from the lncRNA/circRNA-miRNA-mRNA ceRNA network [8].

However, serum RNAs can often be degraded by RNA enzymes and, thus, may not accurately reflect pathological differences; exosomes can protect serum RNAs from degradation [9]. Exosomes are small vesicles with a diameter of approximately 30–150 nm containing proteins, nucleic acids, and lipids [10] and are related to diverse regulatory processes in cardiovascular disorders, including myocardial injury, repair, and regeneration [11].

To better understand the underlying molecular regulatory mechanisms of CHD, we aimed to identify differentially expressed exosomal-related lncRNAs, circRNAs, and miRNAs and used these to construct a ceRNA network to discover accurate and reliable diagnostic biomarkers and therapeutic targets for CHD.

## 2. Materials and Methods

### 2.1. Data Collection

**Figure 1:**
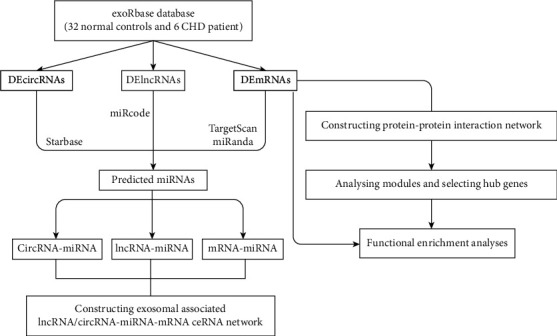
Study flowchart. Abbreviations: CHD, coronary artery disease; mRNAs, messenger RNAs; miRNAs, microRNAs; lncRNAs, long noncoding RNAs; circRNAs, circular RNAs, DEMs, differentially expressed mRNAs; DELs, differentially expressed lncRNAs; DECs, differentially expressed circRNAs; and ceRNA, competing endogenous RNA.

A flowchart of the study is shown in Figure 1. The exoRBase database (http://www.exorbase.org/) is a repository of circRNAs, lncRNAs, and mRNAs derived from RNA-seq data, including analysis of human blood exosomes. These samples come from different biological conditions, including normal people (NP), CHD, colorectal cancer, hepatocellular carcinoma, pancreatic adenocarcinoma, and breast cancer [12]. In this study, data from NP and CHD blood samples were downloaded, including 6 patients with CHD and 32 normal controls.

### 2.2. Identification of Differentially Expressed mRNAs, lncRNAs, and circRNAs

The lists of differentially expressed circRNAs (DECs), lncRNAs (DELs), and mRNAs (DEMs) between controls and patients with CHD were generated using the LIMMA package in *R* software. The values of |log_2_ (fold change (FC))| > 0 and *P* value < 0.05 were selected as cutoff criteria.

### 2.3. Integration of a PPI Network and Module Analysis

A protein-protein interaction (PPI) network of DEMs was constructed by STRING (https://string‐db.org) and visualized with Cytoscape software [13]. Furthermore, the Molecular Complex Detection (MCODE) application in Cytoscape was used to select the PPI network modules, with cutoff = 2, node score cutoff = 0.2, k-core = 2, and maximum depth = 100 as selection criteria. In addition, nodes with degree ≥5 were identified as hub nodes in the PPI network.

### 2.4. Functional Enrichment Analyses

Gene ontology (GO) analysis was used to annotate the DEMs and hub genes based on biological processes (BP), cellular components (CC), and molecular functions (MF) [14]. To investigate the biological function of DEMs and hub genes, the database for annotation, visualization, and integrated discovery (DAVID) online tool (version 6.8; http://david.abcc.ncifcrf.gov) was utilized to perform GO analysis [15]. In addition, the KOBAS 3.0 online analysis database was used to perform pathway enrichment analysis [16]. The significant enrichment threshold for GO and KEGG (Kyoto Encyclopedia of Genes and Genomes) analyses was a *P* value <0.05 and count ≥ 2.

### 2.5. Prediction of microRNAs Targeting mRNAs

The TargetScan (http://www.targetscan.org/vert_72/) [17] and miRanda (http://www.mirdb.org/) [18] databases were used to predict miRNAs that target the DEMs. To increase the accuracy of the predictions, the targeting miRNAs predicted by both databases were used. The miRcode (http://www.mircode.org/) [19] and starBase (http://starbase.sysu.edu.cn/) [20] databases were used to predict miRNAs targeted by the DELs and DECs, respectively. Pairs of miRNAs-mRNAs, lncRNA-miRNA, and circRNA-miRNA were subsequently constructed.

### 2.6. Construction of the lncRNA/circRNA-miRNA-mRNA ceRNA Network

CeRNA regulation has been reported to serve important roles in human disease; the circRNA or lncRNA-miRNA-mRNA interaction network was constructed to explore the associations among circRNAs, lncRNAs, miRNAs, and mRNAs [21]. Finally, Cytoscape was used to visualize the lncRNA/circRNA-miRNA-mRNA network.

### 2.7. Statistical Analysis

All data were expressed as the mean ± standard error. Statistical analyses were performed using GraphPad Prism 6 (GraphPad Software, Inc., La Jolla, CA, USA). *P* < 0.05 was considered to indicate a statistically significant difference.

## 3. Results

### 3.1. Differential Expression Analysis

**Figure 2:**
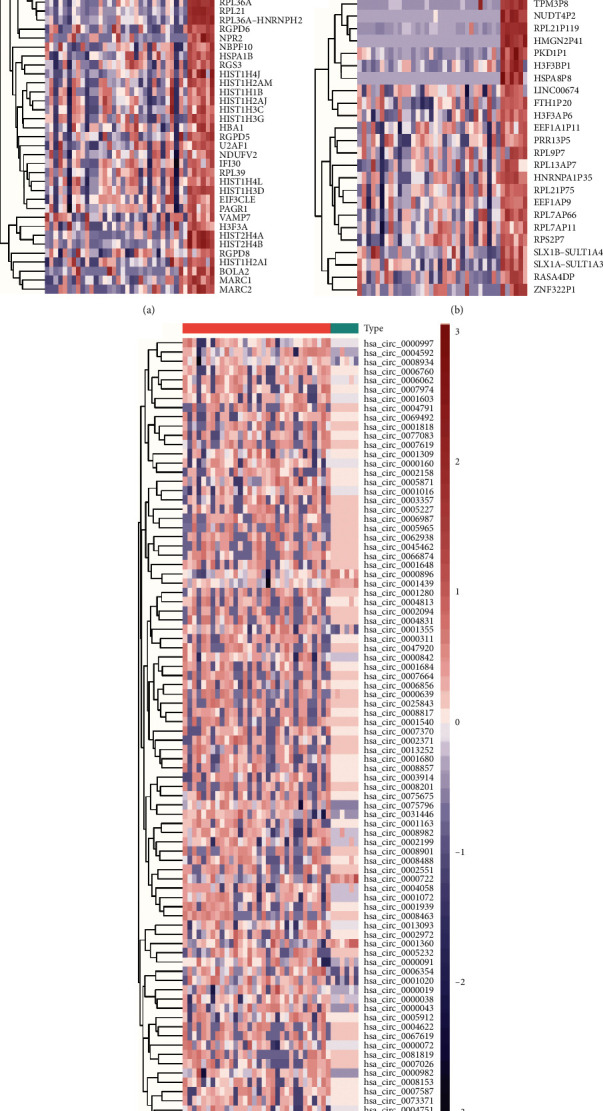
Hierarchical clustering and heat map analysis of differentially expressed profiles of exosomal RNAs. Note: (a) mRNAs, (b) lncRNAs, and (c) circRNAs. The color scale indicates the expression of differentially expressed exosomal RNAs. Red and blue indicate upand downregulation, respectively. Abbreviations are the same as in Figure 1.

A total of 312 DEMs (55 upregulated and 257 downregulated), 43 DELs (24 upregulated and 19 downregulated), and 85 DECs (4 upregulated and 81 downregulated) were identified between the CHD patients and control individuals. The complete upregulated and downregulated DEMs, DELs, and DECs are listed in supplementary materials: Tables S1–S3. Finally, based on the chosen criteria of *P* value <0.05 and | log_2_(FC) | > 0.5, we plotted the heat maps for the DEMs, DELs, and DECs, respectively, as shown in Figures 2(a)–2(c).

### 3.2. PPI Network and Module Analyses

**Figure 3:**
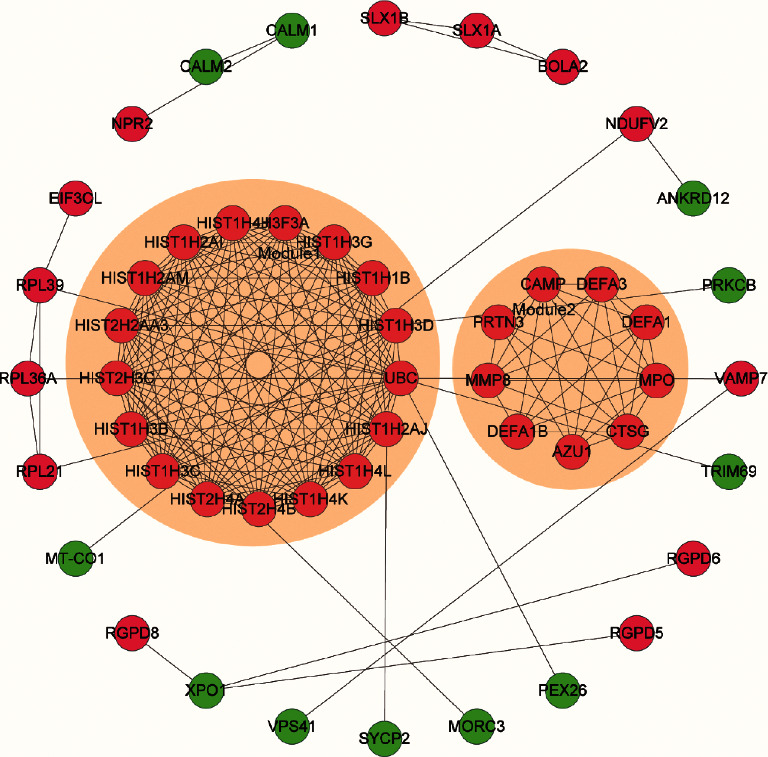
Protein-protein interaction network and the significant modules. Note: the red nodes represent the upregulated mRNAs and the green nodes represent the downregulated mRNAs. The most significant module identified by MCODE had a score = 16.875; the second most significant module score = 7.429. Abbreviations: MCODE: molecular complex detection; other abbreviations are the same as in Figure 1.

To further study the specific DEMs from an interactive perspective, a PPI network was constructed using the STRING database. A statistically significant PPI network consisted of 50 nodes and 189 edges. Nodes with a degree of ≥5 were regarded as hub mRNAs in the network. Two modules were formed in the PPI network with an MCODE score ≥7: module 1 with an MCODE score of 16.875 (nodes = 17) and module 2 with an MCODE score of 7.429 (nodes = 8). Hub mRNAs, namely, ubiquitin C (*UBC*) and 16 histone cluster family genes, were present in module 1, and cathepsin *G*, myeloperoxidase, cathelicidin antimicrobial peptide, defensin alpha 1, defensin alpha 3, matrix metallopeptidase 8, azurocidin 1, and defensin alpha 1B were present in module 2 (Figure 3).

### 3.3. Functional Enrichment Analyses

**Figure 4:**
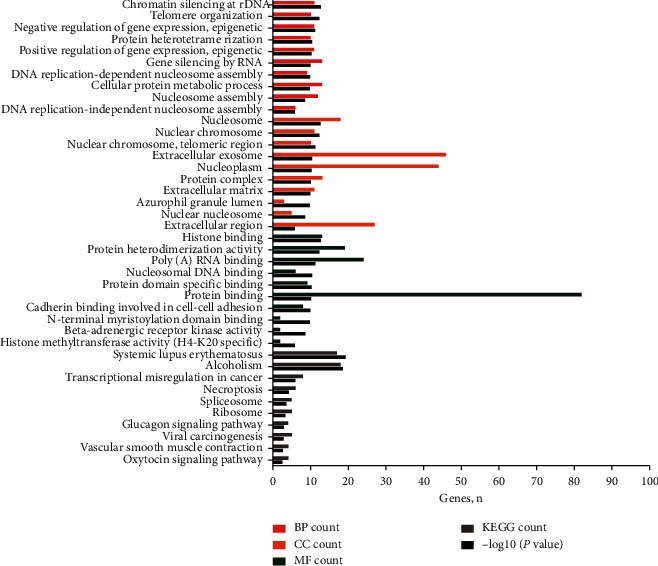
The functional terms and pathways enriched for the differentially expressed mRNAs. Note: the red lines represent biological processes, the orange lines represent cellular components, the green lines represent molecular functions, the black lines represent KEGG pathways, and the gray lines represent −log10 (*P* value). Abbreviation: KEGG, Kyoto Encyclopedia of Genes and Genomes.

**Figure 5:**
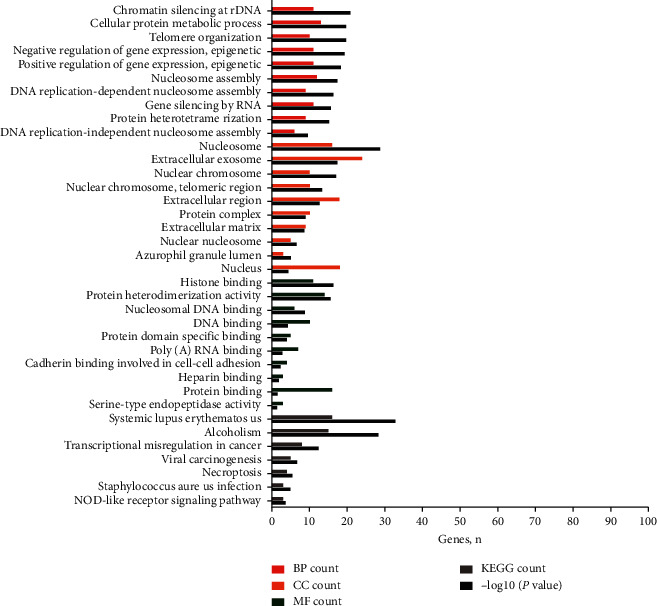
Functional and pathway enrichment analyses of hub genes selected from the protein-protein interaction network. Note: the red lines represent biological processes, the orange lines represent cellular components, the green lines represent molecular functions, the black lines represent KEGG pathways, and the gray lines represent −log10 (*P* value). Abbreviations are the same as in Figure 4.

Functional enrichment analyses indicated that the DEMs were mainly enriched in “chromatin silencing at rDNA,” “telomere organization,” and “negative regulation of gene expression, epigenetic” for the BPs. CC analysis showed that the DEMs were significantly enriched for the “nucleosome,” “nuclear chromosome,” and “nuclear chromosome” components. For the MF category, the DEMs were enriched in “histone binding,” “protein heterodimerization activity,” and “poly(A) RNA binding” (Figure 4 and supplementary materials: Table S4). The enrichment analyses for hub genes showed that the biological function of UBC was enriched for a “cellular protein metabolic process,” its location was greatly enriched within the “extracellular exosome” compartment, and its molecular function was “poly(A) RNA binding” (Figure 5 and supplementary materials: Table S5).

### 3.4. LncRNA/circRNA-miRNA-mRNA ceRNA Network

**Figure 6:**
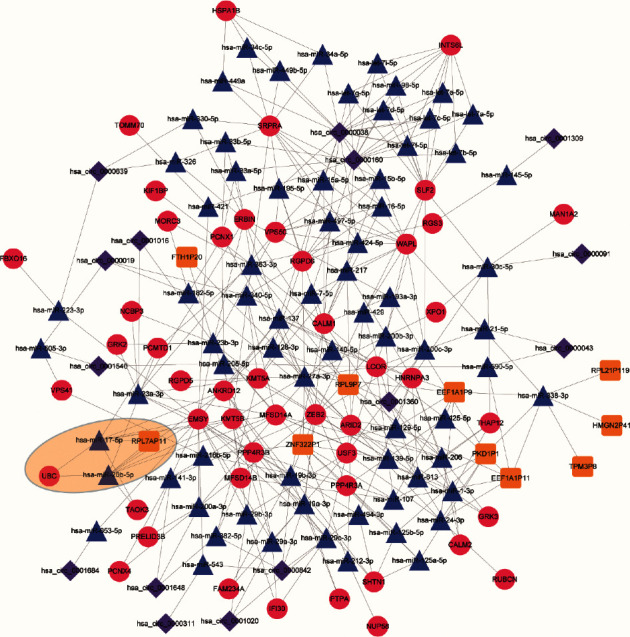
Competing endogenous RNA interaction network. Note: the red circle represents mRNAs, blue triangle represents miRNAs, brown hexagon represents lncRNAs, and purple diamond represents circRNAs. Abbreviations are the same as in Figure 1.

Using the several construction tools described above, 175, 166, and 171 miRNAs were predicted to target DEMs, DELs, and DECs, respectively. The Venn diagram of predicted miRNAs is shown in supplementary materials: Figure S1. Subsequently, ceRNA network analyses were performed to unravel the functions of the identified differentially expressed exosomal-associated ceRNA network in CHD patients. This ceRNA network consisted of 48 DEMs, 73 predicted miRNAs, 10 DELs, and 15 DECs. From the ceRNA network, we identified lncRNA RPL7AP11 as competing for binding to hsa-miR-20a-5p and hsa-miR-17-5p, thereby affecting *UBC* expression (Figure 6 and supplementary materials: Table S6). These results suggest that the ceRNA networks we predict in this paper might be a key factor underlying the pathogenesis of CHD.

## 4. Discussion

Atherosclerotic disease and its thrombotic complications may lead to the development of CHD and, if untreated, can progress to myocardial infarction. Exosomes are a type of extracellular vesicle that contain molecular constituents (protein, DNA, and RNA) of the cells that secrete them [22].

Recently, dysregulated expression of RNAs (lncRNAs, circRNAs, miRNAs, and mRNAs) and regulation of their networks have been confirmed to affect the pathogenesis and progression of various tumors, such as endometrial carcinoma [23], ovarian cancer [24], and ependymoma [25]. In the cardiovascular system, several novel serum RNAs have been found to be associated with CHD [8, 26]. Although serum RNAs are often degraded by RNA enzymes and might not accurately reflect pathological differences, exosomes can protect them from degradation [9]. We, therefore, identified serum exosomal-associated RNAs and constructed a ceRNA network associated with CHD, revealing a new targeting axis in its pathogenesis. To our knowledge, this is the first study to explore exosomal-associated ceRNA networks in CHD.

In this study, we first identified 312 DEMs, 85 DECs, and 43 DELs involved in the pathogenesis of coronary heart disease. Enrichment analysis and PPI network construction were subsequently performed and revealed UBC to be one of the most important hub genes. After predicting miRNAs targeting mRNA, an exosomal-associated circRNA/lncRNA-miRNA-mRNA ceRNA network was constructed. Our results suggest specific ceRNA axes in the pathogenesis of CHD that may be promising targets for diagnosis of CHD.

UBC belongs to the ubiquitin family and is associated with protein degradation, DNA repair, kinase modification, autophagy, regulation of inflammation, and cell signaling pathways [27, 28]. Our enrichment analysis of both DEMs and hub genes showed that UBC participates in cellular protein metabolic processes. Ji et al. [29] showed that the expression of ubiquitin was significantly higher in CHD patients than in healthy individuals and the levels of ubiquitin varied with the severity of different classes of CHD. Our study further confirmed the function of UBC in the pathogenesis of CHD, suggesting its potential value as a noninvasive biomarker.

MiR-17-5p has been reported to regulate the cell cycle, proliferation, and apoptosis, and broad support has been provided for its role in regulation of cardiovascular diseases. Deletion of miR17 in neonatal mice is lethal, and overexpression of miR-17-5p can extend the life span of mice [30]. Liu et al. [31] showed that upregulation of miR-17-5p could contribute to hypoxia-induced proliferation of human pulmonary artery smooth muscle cells, leading to pulmonary hypertension. Yang et al. [32] found that miR-17-5p silencing protects heart function after acute myocardial infarction by decreasing the rate of apoptosis and repairing vascular injury. Moreover, recent studies have shown that circulating miR-17-5p could be a novel biomarker for diagnosis of acute myocardial infarction [33].

MiR-20b-5p has been shown to attenuate hypoxia-induced apoptosis in cardiomyocytes [34]. Also, Zhen et al. [35] found that overexpression of miR-20b-5p could increase cell viability and repress autophagy and apoptosis in human umbilical vein endothelial cells that had experienced hypoxia-reoxygenation injury. Since both hypoxia and hypoxia-reoxygenation models are similar to patients with myocardial infarction and subsequent revascularization, miR-20b-5p may play a role in regulating CHD. However, there has been little research on the function of miR-20b-5p in CHD patients, and confirmation of its role requires further research.

The lncRNA RPL7AP11 (ribosomal protein L7a pseudogene 11) is a pseudogene of ribosomal protein L7a (RPL7A). Zhang et al. [36] found that RPL7A was downregulated in a vascular endothelial cell line (ECV 304) induced by high-density lipoprotein. Pseudogenes, abundant in the human genome, were traditionally considered to be nonfunctional “junk” genes [37], although recent studies have demonstrated their role in various diseases. However, there has been limited research on RPL7AP11, and more evidence is needed.

Our study has shown that RPL7AP11 can sponge hsa-miR-17-5p and hsa-miR-20b-5p to upregulate UBC, thus regulating the pathogenesis of CHD through cellular protein metabolism.

There are several limitations to the present study. Firstly, the sample was not large. An additional validation cohort should be included in future studies to analyze the expression of these identified lncRNAs, circRNAs, miRNAs, and mRNAs. Secondly, how these novel exosomal-associated ceRNA axes participate in the development of CHD is still unclear. Further cell and animal experiments are needed to verify these findings. Moreover, further studies should focus on exploring the ceRNA networks between lncRNA/circRNA in serum exosomes and miRNA-mRNA in target recipient cells.

## 5. Conclusions

In conclusion, our comprehensive study identified several exosomal-associated lncRNA/circRNA-miRNA-mRNA interaction axes (RPL7AP11/hsa-miR-17-5p/UBC and RPL7AP11/hsa-miR-20b-5p/UBC) in the progression of CHD, which may be crucial targets for disease treatment.

## Data Availability

The authors confirm that all data underlying the findings are fully available without restriction. All relevant data are accessible from the exoRBase database (http://www.exorbase.org/). Processed data are available from the corresponding author Jing Wang (e-mail: roubaobao09@163.com).

## Supplementary Materials

Supplementary Materials Table S1: all upregulated and downregulated DEMs between the normal controls and the CHD samples. Table S2: all upregulated and downregulated DELs between the normal controls and the CHD samples. Table S3: all upregulated and downregulated DECs between the normal controls and the CHD samples. Table S4: enrichment analyses of differentially expressed mRNAs. Table S5: enrichment analyses of module hub genes. Table S6: complete list of competing endogenous RNA network pairs. Figure S1: Venn diagram of predicted microRNAs targeting DEMs, DELs, and DECs. Abbreviations: DEMs, differentially expressed mRNAs; DELs, differentially expressed lncRNAs; and DECs, differentially expressed circRNAs.
