# Supplementary material for: Identifying a Serum Exosomal-Associated lncRNA/circRNA-miRNA-mRNA Network in Coronary Heart Disease
Source: Cardiol Res Pract. 2021 Jun 23;2021:6682183. doi: 10.1155/2021/6682183 (PMC8249161; doi:10.1155/2021/6682183)

**Supplementary Materials**

**Table S1.** All upregulated and downregulated DEMs between the normal controls and the CHD samples.

| mRNAs | logFC | *P*-Value |
| --- | --- | --- |
| ERBIN | -0.370589998 | 6.00E-15 |
| RPL21 | 0.951681075 | 3.08E-09 |
| SLF1 | -0.450528559 | 1.99E-08 |
| RPL36A | 1.0730301 | 2.83E-08 |
| RPL36A-HNRNPH2 | 0.970017946 | 3.55E-08 |
| ARHGAP45 | -0.440353334 | 7.68E-08 |
| HIST1H3D | 0.982013909 | 1.04E-07 |
| GRK3 | -0.437481046 | 1.18E-07 |
| NRDC | -0.43362202 | 2.17E-07 |
| EMSY | -0.424368217 | 1.15E-06 |
| AC005943.2 | -0.421362907 | 2.12E-06 |
| SLC25A6 | 1.515313583 | 6.73E-06 |
| AF011889.5 | -0.413796956 | 1.16E-05 |
| AL109923.1 | -0.412564694 | 1.56E-05 |
| USF3 | -0.411572208 | 1.99E-05 |
| RGPD6 | 1.248438567 | 2.41E-05 |
| RP11-474G23.1 | -0.410736233 | 2.46E-05 |
| CTD-3214H19.4 | -0.408975451 | 3.86E-05 |
| PPP4R3B | -0.408939326 | 3.90E-05 |
| SRPRA | -0.408133071 | 4.81E-05 |
| CTD-2545G14.7 | -0.407668365 | 5.43E-05 |
| RP11-231C18.3 | -0.407447219 | 5.76E-05 |
| SLF2 | -0.406779275 | 6.88E-05 |
| RP11-111K18.1 | -0.405715579 | 9.16E-05 |
| TMX2-CTNND1 | 1.671373048 | 9.30E-05 |
| KMT5B | -0.405098148 | 0.000108351 |
| CTD-2132N18.3 | -0.404677479 | 0.000121534 |
| WAPL | -0.404402016 | 0.000131056 |
| AL078584.1 | -0.404150909 | 0.000140409 |
| RP11-286N22.8 | -0.404088549 | 0.000142836 |
| HSPA1B | 1.075423594 | 0.000151523 |
| TCEAL9 | -0.402735045 | 0.000207656 |
| RP11-302B13.5 | -0.402645103 | 0.000212912 |
| RP11-146B14.1 | -0.402091448 | 0.000248398 |
| RP11-295K3.1 | -0.402021167 | 0.000253315 |
| PTPA | -0.401970632 | 0.000256912 |
| AL513412.1 | -0.401674378 | 0.000279069 |
| CAMP | 1.291241406 | 0.000279313 |
| SPDYA | -0.401467215 | 0.000295708 |
| UTP4 | -0.400828429 | 0.000353624 |
| CARNMT1 | -0.400706416 | 0.000365929 |
| AC073610.5 | -0.400450096 | 0.000393203 |
| THAP12 | -0.399709693 | 0.000484026 |
| PPP4R3A | -0.399444027 | 0.000521509 |
| RP11-561B11.2 | -0.399042394 | 0.000583747 |
| SNU13 | -0.398903449 | 0.000606957 |
| RP11-2C24.9 | -0.398876779 | 0.000611516 |
| SVBP | -0.398777675 | 0.000628756 |
| NCBP3 | -0.398647937 | 0.000652057 |
| VPS50 | -0.398563993 | 0.000667587 |
| RP5-1028K7.3 | -0.398469742 | 0.000685461 |
| RP11-512M8.5 | -0.398419181 | 0.000695244 |
| RP11-507K12.1 | -0.398129108 | 0.000754095 |
| SMIM10L1 | -0.397807187 | 0.000825169 |
| RP11-542C16.2 | -0.397732269 | 0.000842632 |
| GRK2 | -0.397689675 | 0.000852723 |
| RP11-73M18.2 | -0.397385262 | 0.000928368 |
| RP11-426L16.10 | -0.397068094 | 0.001014164 |
| DEFA1B | 2.081303074 | 0.001071531 |
| RP11-468E2.4 | -0.396496593 | 0.001188673 |
| LINS1 | -0.396464631 | 0.001199249 |
| RGS3 | 0.599533722 | 0.001338321 |
| RGPD5 | 0.840483836 | 0.001363471 |
| RP11-529K1.3 | -0.395985451 | 0.00136915 |
| XXbac-BPG32J3.22 | -0.395809222 | 0.001437293 |
| RP11-449H3.3 | -0.395702277 | 0.001480223 |
| NSMCE3 | -0.395689382 | 0.001485482 |
| RP11-437B10.1 | -0.395560455 | 0.001539054 |
| AL356053.1 | -0.39532983 | 0.001639534 |
| RP4-583P15.15 | -0.39531761 | 0.001645029 |
| HIST2H2AA4 | 1.893965869 | 0.001754736 |
| RP11-123K3.4 | -0.394991883 | 0.001798154 |
| AC013264.1 | -0.394797296 | 0.001896002 |
| HIST1H4K | 1.680786924 | 0.001930316 |
| TOMM70 | -0.394653053 | 0.001971772 |
| DEFA3 | 1.755758484 | 0.002002426 |
| EFL1 | -0.394591241 | 0.002005111 |
| NUP58 | -0.394482675 | 0.002064961 |
| U2AF1 | 0.796415505 | 0.002167563 |
| MPO | 1.381699381 | 0.002221902 |
| DEFA1 | 1.743859344 | 0.002243182 |
| ABHD18 | -0.394157083 | 0.00225472 |
| AC007192.4 | -0.39409527 | 0.00229255 |
| AZU1 | 1.562939612 | 0.002377994 |
| AC068533.7 | -0.393912724 | 0.002407775 |
| MMP8 | 1.475244309 | 0.002746863 |
| PCNX1 | -0.393401882 | 0.002759652 |
| RP11-307N16.6 | -0.393378246 | 0.002777038 |
| CTC-432M15.3 | -0.393343745 | 0.002802599 |
| HIST2H4B | 1.932197468 | 0.002846407 |
| RPL39 | 0.587682725 | 0.002907319 |
| HIST2H4A | 1.839453979 | 0.002965435 |
| TRIM69 | -0.383669176 | 0.003005885 |
| CTD-2207O23.3 | -0.393017798 | 0.003055071 |
| RP11-762I7.5 | -0.392968223 | 0.003095263 |
| CTD-2370N5.3 | -0.392942333 | 0.003116446 |
| RP11-468E2.1 | -0.392912516 | 0.003141008 |
| RP5-1021I20.4 | -0.392893825 | 0.003156495 |
| RP11-49K24.6 | -0.392877657 | 0.003169948 |
| AC127070.1 | -0.392825279 | 0.003213896 |
| RUBCN | -0.392756745 | 0.003272246 |
| AL928654.7 | -0.392730409 | 0.003294927 |
| RP11-574F21.3 | -0.392627883 | 0.003384603 |
| RP4-635E18.9 | -0.392564026 | 0.003441582 |
| DCAF1 | -0.392549416 | 0.003454742 |
| KMT5A | -0.392494129 | 0.003504957 |
| GCN1 | -0.39237854 | 0.003612107 |
| RP11-864I4.1 | -0.392162124 | 0.003820794 |
| SHTN1 | -0.391901039 | 0.004087115 |
| ARPC4-TTLL3 | -0.245489778 | 0.004125996 |
| FAAP20 | -0.391851624 | 0.004139374 |
| RACK1 | -0.391653758 | 0.004354732 |
| AP000275.65 | -0.391590138 | 0.004426092 |
| CTD-2116N17.1 | -0.391543444 | 0.004479134 |
| PUM3 | -0.391452534 | 0.004584045 |
| RP11-1035H13.3 | -0.391410043 | 0.004633832 |
| RP11-849F2.7 | -0.391389413 | 0.004658178 |
| AP000304.12 | -0.391305709 | 0.004758144 |
| CTB-50L17.10 | -0.391289076 | 0.004778235 |
| AC009133.23 | -0.391217148 | 0.004865995 |
| RP11-244H3.4 | -0.391112391 | 0.004996384 |
| CTC-435M10.3 | -0.391053584 | 0.005070937 |
| RP11-382A20.3 | -0.391023125 | 0.005109941 |
| AL513122.2 | -0.391012244 | 0.005123938 |
| AL513122.1 | -0.39099598 | 0.005144924 |
| AC104534.3 | -0.390936685 | 0.005222084 |
| RP11-507M3.1 | -0.390931748 | 0.005228554 |
| CTD-2192J16.20 | -0.390789849 | 0.005417599 |
| RP11-644F5.10 | -0.390726055 | 0.005504544 |
| H3F3A | 0.529859299 | 0.005592531 |
| CNOT9 | -0.39042043 | 0.005938396 |
| HIST2H3C | 2.158798538 | 0.005971123 |
| 1-Mar | 1.522615173 | 0.006030256 |
| AC096949.1 | -0.390358076 | 0.006030517 |
| RP11-77K12.1 | -0.390357877 | 0.006030812 |
| RP5-864K19.6 | -0.390341745 | 0.00605485 |
| LCOR | -0.31916781 | 0.006120601 |
| HIST1H2AM | 0.879249693 | 0.006298744 |
| CTC-260F20.3 | -0.390135224 | 0.006370074 |
| AC009950.1 | -0.390113989 | 0.006403286 |
| RP11-407N17.3 | -0.390078229 | 0.006459556 |
| PRKCB | -0.136275488 | 0.006497622 |
| HIST1H3G | 1.166933499 | 0.00649766 |
| MFSD14A | -0.390029381 | 0.006537117 |
| PRELID3B | -0.389898103 | 0.006749589 |
| LLfos-48D6.2 | -0.389776621 | 0.006951524 |
| RP11-111H13.1 | -0.389728361 | 0.007033185 |
| RP11-96O20.4 | -0.389654528 | 0.007159725 |
| CTSG | 1.660791204 | 0.007190844 |
| RP11-314N13.10 | -0.389600253 | 0.007253994 |
| AC090498.1 | -0.389562952 | 0.007319401 |
| TMEM94 | -0.389409029 | 0.007594702 |
| 2-Mar | 1.735208542 | 0.007631725 |
| RP5-850E9.3 | -0.389338742 | 0.007723344 |
| CTD-2278I10.6 | -0.389241763 | 0.007903902 |
| RP1-34B20.21 | -0.389145257 | 0.008087152 |
| CTD-3138B18.4 | -0.389119671 | 0.008136339 |
| FCMR | -0.389092746 | 0.008188374 |
| AL358113.1 | -0.389052567 | 0.008266554 |
| RP11-12J10.3 | -0.389020404 | 0.008329592 |
| AC013461.1 | -0.388862035 | 0.008645984 |
| CTD-2369P2.12 | -0.388846527 | 0.008677507 |
| RP11-152F13.10 | -0.388829783 | 0.00871165 |
| HIST2H2AA3 | 2.077766629 | 0.008869749 |
| HIST1H4L | 0.64941352 | 0.008871405 |
| CTC-487M23.8 | -0.388658668 | 0.009067149 |
| RP11-463D19.2 | -0.388534189 | 0.009333377 |
| RP11-697E2.6 | -0.388508614 | 0.009388881 |
| AC010642.1 | -0.388367686 | 0.009699721 |
| RP11-176H8.1 | -0.388347687 | 0.009744523 |
| RP11-343C2.11 | -0.388019329 | 0.010505145 |
| AC027682.1 | -0.387851908 | 0.010911537 |
| RP4-559A3.7 | -0.387814991 | 0.011002873 |
| INTS6L | -0.387798844 | 0.011043017 |
| RP11-552F3.12 | -0.387778018 | 0.011094976 |
| RP11-411B6.6 | -0.38777019 | 0.011114559 |
| PRICKLE4 | -0.383103833 | 0.011297262 |
| RP11-438J1.1 | -0.387501682 | 0.011803621 |
| BOLA2 | 1.589614934 | 0.011911454 |
| EIF3CL | 0.780422824 | 0.012263451 |
| RP11-598P20.5 | -0.387283784 | 0.012388119 |
| RP11-701H24.9 | -0.387168731 | 0.0127061 |
| BORCS6 | -0.387119117 | 0.012845244 |
| RP11-212D19.4 | -0.386957408 | 0.013307322 |
| CTB-133G6.1 | -0.386920507 | 0.013414617 |
| RP11-295P9.13 | -0.386890679 | 0.013501852 |
| CTD-2410N18.5 | -0.386864525 | 0.013578719 |
| NPR2 | 1.790283597 | 0.013804357 |
| AC026348.1 | -0.386543219 | 0.01455189 |
| ERVK3-1 | -0.386539675 | 0.014562927 |
| CRAMP1 | -0.386494309 | 0.014704774 |
| AL022067.1 | -0.386478457 | 0.014754595 |
| RP11-33O4.2 | -0.386463966 | 0.014800259 |
| KAT14 | -0.386378345 | 0.01507234 |
| SNX6 | -0.135030092 | 0.015244541 |
| ZBED1 | 1.94088809 | 0.01547668 |
| PCMTD1 | -0.191517637 | 0.015485627 |
| BBOF1 | -0.386221552 | 0.015580812 |
| HIST1H3B | 0.914957324 | 0.015722611 |
| JAML | -0.386169082 | 0.015753951 |
| HIST1H2AI | 0.744686139 | 0.015802168 |
| XXbac-BPG32J3.20 | -0.38763255 | 0.015870323 |
| CTD-2192J16.22 | -0.386085778 | 0.016031925 |
| IFI30 | 0.60321534 | 0.016112035 |
| CALM1 | -0.118828654 | 0.016350414 |
| QTRT2 | -0.385886443 | 0.016712654 |
| PRUNE1 | -0.385866957 | 0.016780386 |
| PCNX3 | -0.385802186 | 0.017007059 |
| RP11-793H13.10 | -0.385668397 | 0.017482776 |
| FBXO16 | -0.385597935 | 0.01773741 |
| AC023283.1 | -0.385597813 | 0.017737853 |
| RP11-574K11.31 | -0.385578157 | 0.017809396 |
| ANKRD12 | -0.176038532 | 0.018564665 |
| RP11-330H6.5 | -0.385303174 | 0.018833643 |
| GPAT4 | -0.385180789 | 0.019303688 |
| NBPF10 | 0.943583296 | 0.01957151 |
| UBC | 0.319510841 | 0.020316527 |
| RCC1L | -0.384869336 | 0.020539957 |
| AC069368.3 | -0.384831977 | 0.020692154 |
| SGF29 | -0.384802314 | 0.020813597 |
| AL034550.1 | -0.384793296 | 0.020850622 |
| NDUFV2 | 0.544322886 | 0.021610995 |
| COPS9 | -0.384589607 | 0.021700105 |
| UTP11 | -0.384462772 | 0.022241903 |
| JCHAIN | -0.38434219 | 0.022766192 |
| AL021546.6 | -0.384315478 | 0.022883558 |
| KYAT3 | -0.384209838 | 0.023352068 |
| RP11-691N7.6 | -0.384199396 | 0.023398757 |
| PYM1 | -0.384190069 | 0.023440515 |
| RGPD8 | 0.613719987 | 0.023501986 |
| HIST1H4J | 1.811921223 | 0.02381204 |
| RP13-279N23.2 | -0.383955814 | 0.024507298 |
| TAOK3 | -0.205257704 | 0.025185874 |
| PAGR1 | 0.788233608 | 0.025590637 |
| CTC-479C5.12 | -0.383715949 | 0.025635641 |
| RP11-156P1.2 | -0.383697623 | 0.025723357 |
| ZEB2 | -0.153139328 | 0.026020415 |
| AC113404.1 | -0.383577299 | 0.026304628 |
| AC024592.12 | -0.383568385 | 0.026348063 |
| XXbac-BPG181M17.5 | -0.38353476 | 0.026512358 |
| RP11-51F16.8 | -0.3835315 | 0.026528324 |
| HIST1H3C | 0.908397935 | 0.026745435 |
| RP11-603J24.9 | -0.38347888 | 0.026787003 |
| CARMIL2 | -0.383409476 | 0.027130931 |
| RP11-613M10.9 | -0.383386748 | 0.027244231 |
| RP11-745O10.4 | -0.383248189 | 0.027942228 |
| AC090094.1 | -0.383245232 | 0.027957261 |
| CTD-2550O8.5 | -0.383224978 | 0.028060375 |
| RP11-315D16.2 | -0.38320225 | 0.028176404 |
| RP11-729L2.2 | -0.382979957 | 0.029329014 |
| KIF1BP | -0.382973608 | 0.029362413 |
| RP1-37E16.12 | -0.382967237 | 0.029395951 |
| MRM2 | -0.382926996 | 0.029608397 |
| AC010547.9 | -0.382891444 | 0.029796979 |
| CTD-2021K4.1 | -0.382862288 | 0.02995225 |
| CTD-2545M3.6 | -0.382850306 | 0.030016226 |
| BANK1 | -0.244554545 | 0.030084117 |
| U2AF1L5 | -0.382747655 | 0.030568171 |
| TKFC | -0.382639663 | 0.031156343 |
| NEPRO | -0.382617354 | 0.031278811 |
| ARID2 | -0.190192797 | 0.031532357 |
| RP11-618P17.4 | -0.382375811 | 0.032625886 |
| FAM234A | -0.382793738 | 0.03306602 |
| MCRIP1 | -0.382292564 | 0.033099129 |
| LUZP6 | -0.382356562 | 0.033348348 |
| CALM2 | -0.177227746 | 0.033900384 |
| MORC3 | -0.17979521 | 0.034115797 |
| NAXE | -0.382109168 | 0.034157983 |
| RP11-458D21.5 | -0.38207381 | 0.034364709 |
| AP003419.11 | -0.382003536 | 0.034778049 |
| RP11-108K14.8 | -0.383593991 | 0.035061958 |
| HIST1H1B | 0.857552464 | 0.035113181 |
| RP11-20I23.3 | -0.381941943 | 0.03514304 |
| MAN1A2 | -0.183028284 | 0.035179019 |
| RP5-972B16.2 | -0.381900483 | 0.035390152 |
| HIST1H2AJ | 0.823196379 | 0.035458278 |
| PCNX4 | -0.381864471 | 0.035605729 |
| S100A6 | 0.308087436 | 0.035866111 |
| SLX1B | 1.2438987 | 0.036092545 |
| RP11-140L24.4 | -0.381752779 | 0.036279858 |
| VPS41 | -0.211708964 | 0.036285185 |
| FAM209A | -0.381598002 | 0.03722783 |
| MT-CO1 | -0.381550672 | 0.03752092 |
| AC002985.3 | -0.381750674 | 0.038023047 |
| AC037459.4 | -0.383119156 | 0.038045344 |
| TBC1D26 | -0.381450293 | 0.038147475 |
| SLX1A | 1.731309629 | 0.038956727 |
| LA16c-431H6.6 | -0.38131202 | 0.039021614 |
| BORCS7 | -0.381197059 | 0.039758117 |
| CH17-270A2.2 | -0.38118295 | 0.039849117 |
| HBA1 | 1.240923862 | 0.040371108 |
| XXbac-BPG246D15.9 | -0.38441578 | 0.040545158 |
| TIGAR | -0.381040216 | 0.040777214 |
| CTD-3222D19.2 | -0.381966348 | 0.041216807 |
| RP11-468E2.2 | -0.380662566 | 0.043298451 |
| HPF1 | -0.381073167 | 0.043355993 |
| AL135791.1 | -0.385716328 | 0.043359874 |
| BEST1 | -0.366400504 | 0.043445996 |
| SYCP2 | -0.382376934 | 0.044568297 |
| RP3-468K18.7 | -0.380375813 | 0.045276294 |
| XPO1 | -0.195239031 | 0.045353013 |
| MFSD14B | -0.380444658 | 0.045822817 |
| HNRNPA3 | 0.41310676 | 0.046053976 |
| VAMP7 | 0.748843612 | 0.046079267 |
| STAT | -0.387067102 | 0.04615047 |
| TRMO | -0.380195951 | 0.046544704 |
| PRTN3 | 1.722298924 | 0.047009562 |
| NUDT3 | -0.272978034 | 0.047946086 |
| EEF1AKMT1 | -0.383386209 | 0.048033773 |
| RP11-831H9.11 | -0.379924265 | 0.048501128 |
| AP000295.9 | -0.37990362 | 0.048651781 |
| PEX26 | -0.346080435 | 0.048787379 |

Abbreviations: mRNAs, messenger RNAs; DEMs, differentially expressed mRNAs; CHD, coronary heart disease; FC, fold change.

**Table S2.** All upregulated and downregulated DELs between the normal controls and the CHD samples.

| LncRNAs | logFC | *P-*Value |
| --- | --- | --- |
| RPL21P119 | 1.229767275 | 2.94E-08 |
| FTH1P20 | 0.764782823 | 7.34E-07 |
| RP11-186B7.4 | -0.266405079 | 3.38E-06 |
| RPL21P75 | 0.711897793 | 4.56E-06 |
| TPM3P8 | 1.899306957 | 7.72E-06 |
| RP11-793H13.8 | -0.253360625 | 0.00022079 |
| H3F3AP6 | 0.855660912 | 0.000246817 |
| EEF1A1P9 | 0.896853949 | 0.001301163 |
| HNRNPA1P35 | 1.270060664 | 0.001451018 |
| HMGN2P41 | 2.268718875 | 0.001516821 |
| CTB-89H12.4 | -0.245615002 | 0.001681439 |
| RPS2P7 | 0.890811656 | 0.002478124 |
| RP11-493E12.3 | -0.242803154 | 0.003104427 |
| NUDT4P2 | 2.338495248 | 0.003606308 |
| RPL7AP66 | 1.354724581 | 0.004730372 |
| RP11-120M18.2 | -0.239785412 | 0.005580871 |
| CTD-2562J17.6 | -0.23854315 | 0.006961601 |
| SLX1A-SULT1A3 | 1.484407619 | 0.007542544 |
| RP11-227G15.3 | -0.235947026 | 0.010661646 |
| PRR13P5 | 0.646947058 | 0.012622211 |
| RP11-156P1.3 | -0.234637497 | 0.012992972 |
| RPL7AP11 | 0.757816861 | 0.01686641 |
| CTD-2336O2.1 | -0.23268759 | 0.017099065 |
| AC093642.5 | -0.231724649 | 0.019421566 |
| RPL9P7 | 1.236636156 | 0.019637565 |
| ZNF322P1 | 1.887161643 | 0.022600828 |
| RP11-673C5.1 | -0.230288986 | 0.023260473 |
| H3F3BP1 | 2.060203268 | 0.024319458 |
| EEF1A1P11 | 0.846655873 | 0.02480746 |
| RP11-181C21.4 | -0.229431562 | 0.02577248 |
| RP11-29G8.3 | -0.229260682 | 0.026292933 |
| SLX1B-SULT1A4 | 1.132142885 | 0.027763395 |
| RASA4DP | 1.493869811 | 0.029719338 |
| PKD1P1 | 2.311335982 | 0.031094933 |
| RP11-43N16.4 | -0.227632378 | 0.03158522 |
| GTF2IP4 | -0.227557231 | 0.031844048 |
| LINC00674 | 0.995180458 | 0.03200419 |
| AC007318.5 | -0.227004981 | 0.033785705 |
| RP11-21J18.1 | -0.225799042 | 0.038267133 |
| HSPA8P8 | 2.604178536 | 0.043469546 |
| RPL13AP7 | 1.019061546 | 0.044575003 |
| RP11-206F17.2 | -0.223727693 | 0.046730202 |
| RP11-218M22.1 | -0.223413146 | 0.048098772 |

Abbreviations: lncRNAs, long noncoding RNAs; DELs, differentially expressed lncRNAs; CHD, coronary heart disease; FC, fold change.

**Table S3.** All upregulated and downregulated DECs between the normal controls and the CHD samples.

| circRNAs | logFC | *P-*Value |
| --- | --- | --- |
| hsa_circ_0075796 | -1.068135978 | 2.90E-09 |
| hsa_circ_0000982 | -1.040454847 | 5.21E-06 |
| hsa_circ_0001072 | -1.00917037 | 1.85E-05 |
| hsa_circ_0000842 | -1.007329855 | 2.00E-05 |
| hsa_circ_0031446 | -1.009044201 | 0.000148263 |
| hsa_circ_0000019 | -0.925890811 | 0.000374602 |
| hsa_circ_0000038 | -0.906595291 | 0.001038222 |
| hsa_circ_0000160 | -0.916344945 | 0.001150365 |
| hsa_circ_0006760 | -0.91264654 | 0.001352138 |
| hsa_circ_0008817 | -0.912009913 | 0.001390068 |
| hsa_circ_0007587 | -0.905648204 | 0.001827739 |
| hsa_circ_0001603 | -0.901590408 | 0.002170261 |
| hsa_circ_0000997 | -0.900790094 | 0.002244411 |
| hsa_circ_0073371 | -0.892096548 | 0.003211921 |
| hsa_circ_0001016 | -0.890229163 | 0.003463239 |
| hsa_circ_0000722 | 0.585001479 | 0.004306601 |
| hsa_circ_0006062 | -0.862170272 | 0.005357726 |
| hsa_circ_0001684 | -0.878011733 | 0.005579406 |
| hsa_circ_0003914 | -0.876754412 | 0.005850307 |
| hsa_circ_0013093 | -0.875393853 | 0.006156035 |
| hsa_circ_0001439 | 1.233920667 | 0.006234202 |
| hsa_circ_0004751 | -0.874838061 | 0.006284782 |
| hsa_circ_0001680 | -0.8747713 | 0.0063004 |
| hsa_circ_0000311 | -0.87366925 | 0.006563004 |
| hsa_circ_0077083 | -0.872000542 | 0.006978224 |
| hsa_circ_0008153 | -0.8717593 | 0.007040043 |
| hsa_circ_0001163 | -0.871146295 | 0.007199201 |
| hsa_circ_0006856 | -0.86662308 | 0.008469145 |
| hsa_circ_0004058 | -0.906431743 | 0.010377092 |
| hsa_circ_0007619 | -0.858489761 | 0.011214962 |
| hsa_circ_0001939 | -0.856565628 | 0.011959447 |
| hsa_circ_0005912 | -0.853413806 | 0.013263261 |
| hsa_circ_0000072 | -0.850408295 | 0.014608085 |
| hsa_circ_0006987 | -0.850104828 | 0.014749551 |
| hsa_circ_0002158 | -0.84990526 | 0.01484316 |
| hsa_circ_0069492 | -0.849820991 | 0.014882825 |
| hsa_circ_0001360 | 0.545999488 | 0.015071542 |
| hsa_circ_0001280 | -0.848185511 | 0.015668971 |
| hsa_circ_0008857 | -0.847213517 | 0.016151082 |
| hsa_circ_0002199 | -0.862848909 | 0.016506185 |
| hsa_circ_0001309 | -0.845310903 | 0.017127515 |
| hsa_circ_0008201 | -0.841334593 | 0.019311822 |
| hsa_circ_0004813 | -0.817824797 | 0.020420167 |
| hsa_circ_0005871 | -0.836058422 | 0.022522002 |
| hsa_circ_0013252 | -0.834996388 | 0.023212567 |
| hsa_circ_0004622 | -0.833872688 | 0.023959761 |
| hsa_circ_0007370 | -0.832216423 | 0.025092374 |
| hsa_circ_0062938 | -0.831261425 | 0.025762534 |
| hsa_circ_0007664 | -0.830938737 | 0.025991823 |
| hsa_circ_0075675 | -0.830311473 | 0.02644166 |
| hsa_circ_0002551 | -0.82924078 | 0.027222151 |
| hsa_circ_0001540 | -0.828956663 | 0.027431951 |
| hsa_circ_0000091 | -0.785695684 | 0.027851602 |
| hsa_circ_0067619 | -0.827903224 | 0.028219726 |
| hsa_circ_0008901 | -0.826227457 | 0.029505161 |
| hsa_circ_0045462 | -0.8260623 | 0.029634005 |
| hsa_circ_0002972 | -0.825962982 | 0.029711673 |
| hsa_circ_0047920 | -0.823542879 | 0.031647774 |
| hsa_circ_0002094 | -0.820476847 | 0.034221688 |
| hsa_circ_0005232 | -0.820196927 | 0.034463466 |
| hsa_circ_0006354 | -0.713619106 | 0.034845212 |
| hsa_circ_0004592 | -0.867832846 | 0.035115312 |
| hsa_circ_0008463 | -0.81790139 | 0.036489297 |
| hsa_circ_0008488 | -0.794236124 | 0.037437461 |
| hsa_circ_0007974 | -0.816647494 | 0.0376284 |
| hsa_circ_0003357 | -0.814338366 | 0.039786489 |
| hsa_circ_0004831 | -0.814181003 | 0.039936412 |
| hsa_circ_0066874 | -0.814180037 | 0.039937333 |
| hsa_circ_0001818 | -0.813971145 | 0.040136914 |
| hsa_circ_0000043 | -0.878890848 | 0.040416443 |
| hsa_circ_0001648 | -0.81363628 | 0.040458192 |
| hsa_circ_0081819 | -0.813573435 | 0.040518671 |
| hsa_circ_0000639 | -0.804618436 | 0.040591417 |
| hsa_circ_0005227 | -0.811430645 | 0.042615595 |
| hsa_circ_0007026 | -0.811401738 | 0.042644345 |
| hsa_circ_0008982 | -0.863222949 | 0.042852536 |
| hsa_circ_0000896 | 1.049105046 | 0.043434422 |
| hsa_circ_0005965 | -0.810537788 | 0.043509305 |
| hsa_circ_0025843 | -0.809707465 | 0.044350962 |
| hsa_circ_0076881 | -0.809654566 | 0.044404927 |
| hsa_circ_0008934 | -0.577407165 | 0.044893058 |
| hsa_circ_0004791 | -0.807457603 | 0.046682563 |
| hsa_circ_0001355 | -0.62785776 | 0.046954465 |
| hsa_circ_0001020 | -0.627650214 | 0.047623797 |
| hsa_circ_0002371 | -0.804980177 | 0.049336093 |

Abbreviations: circRNAs, circular RNAs; DECs, differentially expressed circRNAs; CHD, coronary heart disease; FC, fold change.

**Table S4:** Enrichment analyses of differentially expressed mRNAs.

| Identification | Term | Count | *P-*Value |
| --- | --- | --- | --- |
| **GO-BP Term** |  |  |  |
| GO:0000183 | chromatin silencing at rDNA | 11 | 2.33E-13 |
| GO:0032200 | telomere organization | 10 | 4.44E-13 |
| GO:0045814 | negative regulation of gene expression, epigenetic | 11 | 6.29E-12 |
| GO:0051290 | protein heterotetramerization | 10 | 3.82E-11 |
| GO:0045815 | positive regulation of gene expression, epigenetic | 11 | 6.07E-11 |
| GO:0031047 | gene silencing by RNA | 13 | 8.91E-11 |
| GO:0006335 | DNA replication-dependent nucleosome assembly | 9 | 1.27E-10 |
| GO:0044267 | cellular protein metabolic process | 13 | 1.84E-10 |
| GO:0006334 | nucleosome assembly | 12 | 3.03E-09 |
| GO:0006336 | DNA replication-independent nucleosome assembly | 6 | 1.79E-06 |
| GO:0045653 | negative regulation of megakaryocyte differentiation | 5 | 1.12E-05 |
| GO:0019731 | antibacterial humoral response | 6 | 2.63E-05 |
| GO:0016233 | telomere capping | 5 | 3.14E-05 |
| GO:0002227 | innate immune response in mucosa | 5 | 4.43E-05 |
| GO:0050832 | defense response to fungus | 5 | 6.07E-05 |
| GO:0060968 | regulation of gene silencing | 4 | 8.00E-05 |
| GO:0006352 | DNA-templated transcription, initiation | 5 | 1.93E-04 |
| GO:0034080 | CENP-A containing nucleosome assembly | 5 | 3.87E-04 |
| GO:1904837 | beta-catenin-TCF complex assembly | 5 | 3.87E-04 |
| GO:0006342 | chromatin silencing | 5 | 4.61E-04 |
| GO:0051343 | positive regulation of cyclic-nucleotide phosphodiesterase activity | 3 | 6.31E-04 |
| GO:0006303 | double-strand break repair via nonhomologous end joining | 5 | 0.0016489 |
| GO:0006281 | DNA repair | 8 | 0.0029269 |
| GO:0007596 | blood coagulation | 7 | 0.0037346 |
| GO:0006412 | translation | 8 | 0.0043869 |
| GO:0032516 | positive regulation of phosphoprotein phosphatase activity | 3 | 0.0047221 |
| GO:0050830 | defense response to Gram-positive bacterium | 5 | 0.0048941 |
| GO:0031640 | killing of cells of other organism | 3 | 0.0054803 |
| GO:0006974 | cellular response to DNA damage stimulus | 7 | 0.0067359 |
| GO:0036297 | interstrand cross-link repair | 4 | 0.007149 |
| GO:0044130 | negative regulation of growth of symbiont in host | 3 | 0.0071512 |
| GO:0006364 | rRNA processing | 7 | 0.0077012 |
| GO:0000042 | protein targeting to Golgi | 3 | 0.0110877 |
| GO:0035307 | positive regulation of protein dephosphorylation | 3 | 0.0121908 |
| GO:0030520 | intracellular estrogen receptor signaling pathway | 3 | 0.0121908 |
| GO:0010800 | positive regulation of peptidyl-threonine phosphorylation | 3 | 0.0197474 |
| GO:0098609 | cell-cell adhesion | 7 | 0.022344 |
| GO:0030801 | positive regulation of cyclic nucleotide metabolic process | 2 | 0.0239267 |
| GO:1990166 | protein localization to site of double-strand break | 2 | 0.0239267 |
| GO:0010835 | regulation of protein ADP-ribosylation | 2 | 0.0317752 |
| GO:1904431 | positive regulation of t-circle formation | 2 | 0.0317752 |
| GO:0090656 | t-circle formation | 2 | 0.0395612 |
| GO:0034184 | positive regulation of maintenance of mitotic sister chromatid cohesion | 2 | 0.0395612 |
| GO:0006614 | SRP-dependent cotranslational protein targeting to membrane | 4 | 0.040127 |
| GO:0051607 | defense response to virus | 5 | 0.0442414 |
| GO:0010792 | DNA double-strand break processing involved in repair via single-strand annealing | 2 | 0.047285 |
| **GO-CC Term** |  |  |  |
| GO:0000786 | nucleosome | 18 | 1.72E-18 |
| GO:0000228 | nuclear chromosome | 11 | 1.52E-11 |
| GO:0000784 | nuclear chromosome, telomeric region | 10 | 1.29E-06 |
| GO:0070062 | extracellular exosome | 46 | 4.56E-06 |
| GO:0005654 | nucleoplasm | 44 | 0.0000201 |
| GO:0043234 | protein complex | 13 | 0.000158 |
| GO:0031012 | extracellular matrix | 11 | 0.000174 |
| GO:0035578 | azurophil granule lumen | 3 | 0.000399 |
| GO:0000788 | nuclear nucleosome | 5 | 0.000463 |
| GO:0005576 | extracellular region | 27 | 0.000653 |
| GO:0022625 | cytosolic large ribosomal subunit | 5 | 0.0023868 |
| GO:0005634 | nucleus | 62 | 0.0026456 |
| GO:0000790 | nuclear chromatin | 7 | 0.0053024 |
| GO:0005694 | chromosome | 5 | 0.0110954 |
| GO:0005913 | cell-cell adherens junction | 8 | 0.0176383 |
| GO:0034704 | calcium channel complex | 3 | 0.0178356 |
| GO:0033557 | Slx1-Slx4 complex | 2 | 0.0244914 |
| GO:0035577 | azurophil granule membrane | 2 | 0.032522 |
| GO:0089701 | U2AF | 2 | 0.0483868 |
| **GO-MF Term** |  |  |  |
| GO:0042393 | histone binding | 13 | 1.37E-10 |
| GO:0046982 | protein heterodimerization activity | 19 | 1.40E-08 |
| GO:0044822 | poly(A) RNA binding | 24 | 1.21E-05 |
| GO:0031492 | nucleosomal DNA binding | 6 | 2.48E-05 |
| GO:0019904 | protein domain specific binding | 9 | 1.88E-04 |
| GO:0005515 | protein binding | 82 | 0.0065527 |
| GO:0098641 | cadherin binding involved in cell-cell adhesion | 8 | 0.0066452 |
| GO:0031997 | N-terminal myristoylation domain binding | 2 | 0.0225767 |
| GO:0047696 | beta-adrenergic receptor kinase activity | 2 | 0.0299894 |
| GO:0042799 | histone methyltransferase activity (H4-K20 specific) | 2 | 0.0373462 |
| GO:0017108 | 5'-flap endonuclease activity | 2 | 0.0446477 |
| GO:0072542 | protein phosphatase activator activity | 2 | 0.0446477 |
| GO:0003677 | DNA binding | 20 | 0.0482268 |
| **KEGG** |  |  |  |
| hsa05034 | Alcoholism | 18 | 2.48E-19 |
| hsa05322 | Systemic lupus erythematosus | 17 | 5.66E-20 |
| hsa05202 | Transcriptional misregulation in cancer | 8 | 1.34E-06 |
| hsa04217 | Necroptosis | 6 | 6.54E-05 |
| hsa03040 | Spliceosome | 5 | 0.0002702 |
| hsa03010 | Ribosome | 5 | 0.0004706 |
| hsa05203 | Viral carcinogenesis | 5 | 0.0015423 |
| hsa04922 | Glucagon signaling pathway | 4 | 0.0010573 |
| hsa04270 | Vascular smooth muscle contraction | 4 | 0.002303 |
| hsa04921 | Oxytocin signaling pathway | 4 | 0.0038555 |
| hsa03013 | RNA transport | 4 | 0.0050015 |
| hsa04022 | cGMP-PKG signaling pathway | 4 | 0.0052124 |
| hsa05010 | Alzheimer disease | 4 | 0.0056519 |
| hsa04621 | NOD-like receptor signaling pathway | 4 | 0.0064793 |
| hsa04020 | Calcium signaling pathway | 4 | 0.008514 |
| hsa04015 | Rap1 signaling pathway | 4 | 0.0112792 |
| hsa05170 | Human immunodeficiency virus 1 infection | 4 | 0.0116382 |
| hsa04014 | Ras signaling pathway | 4 | 0.0156355 |
| hsa04144 | Endocytosis | 4 | 0.0184019 |
| hsa04010 | MAPK signaling pathway | 4 | 0.0334254 |
| hsa04720 | Long-term potentiation | 3 | 0.0028953 |
| hsa05150 | Staphylococcus aureus infection | 3 | 0.0030141 |
| hsa05031 | Amphetamine addiction | 3 | 0.0030141 |
| hsa05214 | Glioma | 3 | 0.0039304 |
| hsa04971 | Gastric acid secretion | 3 | 0.0039304 |
| hsa04970 | Salivary secretion | 3 | 0.006416 |
| hsa05032 | Morphine addiction | 3 | 0.0066082 |
| hsa04912 | GnRH signaling pathway | 3 | 0.0070027 |
| hsa04713 | Circadian entrainment | 3 | 0.007833 |
| hsa04925 | Aldosterone synthesis and secretion | 3 | 0.0080493 |
| hsa04070 | Phosphatidylinositol signaling system | 3 | 0.008269 |
| hsa04750 | Inflammatory mediator regulation of TRP channels | 3 | 0.0084922 |
| hsa04916 | Melanogenesis | 3 | 0.008719 |
| hsa03008 | Ribosome biogenesis in eukaryotes | 3 | 0.0096614 |
| hsa04724 | Glutamatergic synapse | 3 | 0.0119917 |
| hsa04114 | Oocyte meiosis | 3 | 0.0162058 |
| hsa04728 | Dopaminergic synapse | 3 | 0.0172035 |
| hsa04915 | Estrogen signaling pathway | 3 | 0.0196624 |
| hsa05012 | Parkinson disease | 3 | 0.02115 |
| hsa04218 | Cellular senescence | 3 | 0.0285883 |
| hsa05164 | Influenza A | 3 | 0.0318087 |
| hsa04062 | Chemokine signaling pathway | 3 | 0.0436618 |
| hsa05016 | Huntington disease | 3 | 0.0453492 |
| hsa04744 | Phototransduction | 2 | 0.0065356 |
| hsa00051 | Fructose and mannose metabolism | 2 | 0.0088226 |
| hsa05143 | African trypanosomiasis | 2 | 0.0108724 |
| hsa04340 | Hedgehog signaling pathway | 2 | 0.016808 |
| hsa03460 | Fanconi anemia pathway | 2 | 0.0216108 |
| hsa00310 | Lysine degradation | 2 | 0.0253471 |
| hsa00561 | Glycerolipid metabolism | 2 | 0.02691 |
| hsa05131 | Shigellosis | 2 | 0.0326744 |
| hsa04924 | Renin secretion | 2 | 0.0335342 |
| hsa05133 | Pertussis | 2 | 0.039794 |
| hsa04612 | Antigen processing and presentation | 2 | 0.0407218 |

Abbreviations: GO, gene ontology; BP, biological process; CC, cellular component; MF, molecular function; KEGG, Kyoto Encyclopedia of Genes and Genomes.

**Table S5:** Enrichment analyses of module hub genes.

| Identification | Term | Count | *P-*Value |
| --- | --- | --- | --- |
| **GO-BP Term** |  |  |  |
| GO:0000183 | chromatin silencing at rDNA | 11 | 1.36E-21 |
| GO:0044267 | cellular protein metabolic process | 13 | 2.05E-20 |
| GO:0032200 | telomere organization | 10 | 2.07E-20 |
| GO:0045814 | negative regulation of gene expression, epigenetic | 11 | 3.98E-20 |
| GO:0045815 | positive regulation of gene expression, epigenetic | 11 | 4.12E-19 |
| GO:0006334 | nucleosome assembly | 12 | 3.24E-18 |
| GO:0006335 | DNA replication-dependent nucleosome assembly | 9 | 4.83E-17 |
| GO:0031047 | gene silencing by RNA | 11 | 1.9E-16 |
| GO:0051290 | protein heterotetramerization | 9 | 5.37E-16 |
| GO:0006336 | DNA replication-independent nucleosome assembly | 6 | 2.47E-10 |
| GO:0045653 | negative regulation of megakaryocyte differentiation | 5 | 9.69E-09 |
| GO:0016233 | telomere capping | 5 | 2.79E-08 |
| GO:0050832 | defense response to fungus | 5 | 5.51E-08 |
| GO:0006352 | DNA-templated transcription, initiation | 5 | 1.83E-07 |
| GO:1904837 | beta-catenin-TCF complex assembly | 5 | 3.82E-07 |
| GO:0034080 | CENP-A containing nucleosome assembly | 5 | 3.82E-07 |
| GO:0060968 | regulation of gene silencing | 4 | 4.2E-07 |
| GO:0006303 | double-strand break repair via nonhomologous end joining | 5 | 1.81E-06 |
| GO:0007596 | blood coagulation | 6 | 5.37E-06 |
| GO:0002227 | innate immune response in mucosa | 4 | 5.78E-06 |
| GO:0019731 | antibacterial humoral response | 4 | 3.27E-05 |
| GO:0006342 | chromatin silencing | 4 | 3.5E-05 |
| GO:0031640 | killing of cells of other organism | 3 | 0.000176 |
| GO:0044130 | negative regulation of growth of symbiont in host | 3 | 0.000232 |
| GO:0050830 | defense response to Gram-positive bacterium | 4 | 0.000235 |
| GO:0030520 | intracellular estrogen receptor signaling pathway | 3 | 0.000404 |
| GO:0051607 | defense response to virus | 4 | 0.00162 |
| GO:0045087 | innate immune response | 5 | 0.003001 |
| GO:0098609 | cell-cell adhesion | 4 | 0.006548 |
| GO:0042742 | defense response to bacterium | 3 | 0.018049 |
| GO:0001878 | response to yeast | 2 | 0.018428 |
| GO:0050829 | defense response to Gram-negative bacterium | 2 | 0.075768 |
| GO:0016485 | protein processing | 2 | 0.098052 |
| **GO-CC Term** |  |  |  |
| GO:0000786 | nucleosome | 16 | 1.89E-29 |
| GO:0070062 | extracellular exosome | 24 | 4.02E-18 |
| GO:0000228 | nuclear chromosome | 10 | 9.16E-18 |
| GO:0000784 | nuclear chromosome, telomeric region | 10 | 4.32E-14 |
| GO:0005576 | extracellular region | 18 | 2.13E-13 |
| GO:0043234 | protein complex | 10 | 1.37E-09 |
| GO:0031012 | extracellular matrix | 9 | 2.59E-09 |
| GO:0000788 | nuclear nucleosome | 5 | 3.03E-07 |
| GO:0035578 | azurophil granule lumen | 3 | 9.96E-06 |
| GO:0005634 | nucleus | 18 | 3.78E-05 |
| GO:0000790 | nuclear chromatin | 5 | 1.10E-04 |
| GO:0005615 | extracellular space | 9 | 2.20E-04 |
| GO:0005654 | nucleoplasm | 12 | 3.73E-04 |
| GO:0016020 | membrane | 9 | 0.005492 |
| GO:0005796 | Golgi lumen | 3 | 0.007028 |
| GO:0005913 | cell-cell adherens junction | 4 | 0.008473 |
| GO:0042582 | azurophil granule | 2 | 0.014395 |
| GO:0030141 | secretory granule | 2 | 0.093092 |
| **GO-MF Term** |  |  |  |
| GO:0042393 | histone binding | 11 | 4.61E-17 |
| GO:0046982 | protein heterodimerization activity | 14 | 2.86E-16 |
| GO:0031492 | nucleosomal DNA binding | 6 | 1.81E-09 |
| GO:0003677 | DNA binding | 10 | 5.52E-05 |
| GO:0019904 | protein domain specific binding | 5 | 9.29E-05 |
| GO:0044822 | poly(A) RNA binding | 7 | 0.001516 |
| GO:0098641 | cadherin binding involved in cell-cell adhesion | 4 | 0.004606 |
| GO:0008201 | heparin binding | 3 | 0.015166 |
| GO:0005515 | protein binding | 16 | 0.031494 |
| GO:0004252 | serine-type endopeptidase activity | 3 | 0.036114 |
| **KEGG** |  |  |  |
| hsa05322 | Systemic lupus erythematosus | 16 | 1.59E-33 |
| hsa05034 | Alcoholism | 15 | 4.97E-29 |
| hsa05202 | Transcriptional misregulation in cancer | 8 | 3.09E-13 |
| hsa05203 | Viral carcinogenesis | 5 | 1.85E-07 |
| hsa04217 | Necroptosis | 4 | 3.63E-06 |
| hsa05150 | Staphylococcus aureus infection | 3 | 1.27E-05 |
| hsa04621 | NOD-like receptor signaling pathway | 3 | 0.000206 |

Abbreviations: GO, gene ontology; BP, biological process; CC, cellular component; MF, molecular function; KEGG, Kyoto Encyclopedia of Genes and Genomes.

**Table S6:** Complete list of competing endogenous RNA network pairs.

| mRNA | microRNA | circRNA/lncRNA |
| --- | --- | --- |
| SLF2 | hsa-let-7a-5p | hsa_circ_0000038 |
| INTS6L | hsa-let-7a-5p | hsa_circ_0000160 |
| SLF2 | hsa-let-7a-5p | hsa_circ_0000038 |
| INTS6L | hsa-let-7a-5p | hsa_circ_0000160 |
| SLF2 | hsa-let-7b-5p | hsa_circ_0000038 |
| INTS6L | hsa-let-7b-5p | hsa_circ_0000160 |
| SLF2 | hsa-let-7b-5p | hsa_circ_0000038 |
| INTS6L | hsa-let-7b-5p | hsa_circ_0000160 |
| SLF2 | hsa-let-7c-5p | hsa_circ_0000038 |
| INTS6L | hsa-let-7c-5p | hsa_circ_0000160 |
| SLF2 | hsa-let-7c-5p | hsa_circ_0000038 |
| INTS6L | hsa-let-7c-5p | hsa_circ_0000160 |
| SLF2 | hsa-let-7d-5p | hsa_circ_0000038 |
| INTS6L | hsa-let-7d-5p | hsa_circ_0000160 |
| SLF2 | hsa-let-7d-5p | hsa_circ_0000038 |
| INTS6L | hsa-let-7d-5p | hsa_circ_0000160 |
| SLF2 | hsa-let-7e-5p | hsa_circ_0000038 |
| INTS6L | hsa-let-7e-5p | hsa_circ_0000160 |
| SLF2 | hsa-let-7e-5p | hsa_circ_0000038 |
| INTS6L | hsa-let-7e-5p | hsa_circ_0000160 |
| SLF2 | hsa-let-7f-5p | hsa_circ_0000038 |
| INTS6L | hsa-let-7f-5p | hsa_circ_0000160 |
| SLF2 | hsa-let-7f-5p | hsa_circ_0000038 |
| INTS6L | hsa-let-7f-5p | hsa_circ_0000160 |
| SLF2 | hsa-let-7g-5p | hsa_circ_0000038 |
| INTS6L | hsa-let-7g-5p | hsa_circ_0000160 |
| SLF2 | hsa-let-7g-6p | hsa_circ_0000038 |
| INTS6L | hsa-let-7g-5p | hsa_circ_0000160 |
| SLF2 | hsa-let-7i-5p | hsa_circ_0000038 |
| INTS6L | hsa-let-7i-5p | hsa_circ_0000160 |
| SLF2 | hsa-let-7i-5p | hsa_circ_0000038 |
| INTS6L | hsa-let-7i-5p | hsa_circ_0000160 |
| LCOR | hsa-miR-107 | ZNF322P1 |
| LCOR | hsa-miR-107 | EEF1A1P11 |
| LCOR | hsa-miR-107 | PKD1P1 |
| LCOR | hsa-miR-107 | EEF1A1P9 |
| PPP4R3B | hsa-miR-107 | ZNF322P1 |
| PPP4R4B | hsa-miR-107 | EEF1A1P11 |
| PPP4R5B | hsa-miR-107 | PKD1P1 |
| PPP4R3B | hsa-miR-107 | EEF1A1P9 |
| MFSD14B | hsa-miR-107 | ZNF322P1 |
| MFSD14B | hsa-miR-107 | EEF1A1P11 |
| MFSD14B | hsa-miR-107 | PKD1P1 |
| MFSD14B | hsa-miR-107 | EEF1A1P9 |
| SHTN1 | hsa-miR-125a-5p | ZNF322P1 |
| SHTN1 | hsa-miR-125a-5p | PKD1P1 |
| PPP4R3A | hsa-miR-125a-5p | ZNF322P1 |
| PPP4R3A | hsa-miR-125a-5p | PKD1P1 |
| SHTN1 | hsa-miR-125b-5p | ZNF322P1 |
| SHTN1 | hsa-miR-125b-5p | PKD1P1 |
| PPP4R3A | hsa-miR-125b-5p | ZNF322P1 |
| PPP4R3A | hsa-miR-125b-5p | PKD1P1 |
| ARID2 | hsa-miR-128-3p | hsa_circ_0001360 |
| RGPD6 | hsa-miR-128-3p | hsa_circ_0001361 |
| CALM1 | hsa-miR-129-5p | ZNF322P1 |
| CALM1 | hsa-miR-129-5p | EEF1A1P11 |
| CALM1 | hsa-miR-129-5p | EEF1A1P9 |
| ZEB2 | hsa-miR-129-5p | ZNF322P1 |
| ZEB2 | hsa-miR-129-5p | EEF1A1P11 |
| ZEB2 | hsa-miR-129-5p | EEF1A1P9 |
| HNRNPA3 | hsa-miR-129-5p | ZNF322P1 |
| HNRNPA3 | hsa-miR-129-5p | EEF1A1P11 |
| HNRNPA3 | hsa-miR-129-5p | EEF1A1P9 |
| LCOR | hsa-miR-137 | hsa_circ_0000038 |
| LCOR | hsa-miR-137 | RPL9P7 |
| EMSY | hsa-miR-137 | hsa_circ_0000038 |
| EMSY | hsa-miR-137 | RPL9P7 |
| PPP4R3A | hsa-miR-137 | hsa_circ_0000038 |
| PPP4R3A | hsa-miR-137 | RPL9P7 |
| ANKRD12 | hsa-miR-137 | hsa_circ_0000038 |
| ANKRD12 | hsa-miR-137 | RPL9P7 |
| HNRNPA3 | hsa-miR-137 | hsa_circ_0000038 |
| HNRNPA3 | hsa-miR-137 | RPL9P7 |
| ERBIN | hsa-miR-137 | hsa_circ_0000038 |
| ERBIN | hsa-miR-137 | RPL9P7 |
| LCOR | hsa-miR-139-5p | ZNF322P1 |
| KMT5A | hsa-miR-139-5p | ZNF322P1 |
| ZEB2 | hsa-miR-139-5p | ZNF322P1 |
| MFSD14A | hsa-miR-1-3p | hsa_circ_0001360 |
| THAP12 | hsa-miR-1-3p | hsa_circ_0001360 |
| ARID2 | hsa-miR-1-3p | hsa_circ_0001360 |
| CALM2 | hsa-miR-1-3p | hsa_circ_0001360 |
| HNRNPA3 | hsa-miR-1-3p | hsa_circ_0001360 |
| GRK3 | hsa-miR-1-3p | hsa_circ_0001360 |
| MFSD14A | hsa-miR-140-5p | EEF1A1P9 |
| ERBIN | hsa-miR-140-5p | EEF1A1P9 |
| MFSD14A | hsa-miR-141-3p | hsa_circ_0001648 |
| EMSY | hsa-miR-141-3p | hsa_circ_0001648 |
| PCNX4 | hsa-miR-141-3p | hsa_circ_0001648 |
| ZEB2 | hsa-miR-141-3p | hsa_circ_0001648 |
| PRELID3B | hsa-miR-141-3p | hsa_circ_0001648 |
| RGS3 | hsa-miR-145-5p | hsa_circ_0001309 |
| WAPL | hsa-miR-15a-5p | hsa_circ_0000038 |
| SRPRA | hsa-miR-15a-5p | hsa_circ_0000038 |
| CALM1 | hsa-miR-15a-5p | hsa_circ_0000038 |
| RGPD6 | hsa-miR-15a-5p | hsa_circ_0000038 |
| VPS50 | hsa-miR-15a-5p | hsa_circ_0000038 |
| WAPL | hsa-miR-15b-5p | hsa_circ_0000038 |
| SRPRA | hsa-miR-15b-5p | hsa_circ_0000038 |
| CALM1 | hsa-miR-15b-5p | hsa_circ_0000038 |
| RGPD6 | hsa-miR-15b-5p | hsa_circ_0000038 |
| VPS50 | hsa-miR-15b-5p | hsa_circ_0000038 |
| WAPL | hsa-miR-16-5p | hsa_circ_0000038 |
| SRPRA | hsa-miR-16-5p | hsa_circ_0000038 |
| CALM1 | hsa-miR-16-5p | hsa_circ_0000038 |
| RGPD6 | hsa-miR-16-5p | hsa_circ_0000038 |
| VPS50 | hsa-miR-16-5p | hsa_circ_0000038 |
| MFSD14A | hsa-miR-17-5p | RPL7AP11 |
| KMT5B | hsa-miR-17-5p | RPL7AP11 |
| EMSY | hsa-miR-17-5p | RPL7AP11 |
| TAOK3 | hsa-miR-17-5p | RPL7AP11 |
| UBC | hsa-miR-17-5p | RPL7AP11 |
| ANKRD12 | hsa-miR-17-5p | RPL7AP11 |
| PCMTD1 | hsa-miR-17-5p | RPL7AP11 |
| ARID2 | hsa-miR-182-5p | hsa_circ_0000019 |
| PCNX1 | hsa-miR-182-5p | hsa_circ_0000019 |
| MORC3 | hsa-miR-182-5p | hsa_circ_0000019 |
| CALM1 | hsa-miR-193a-3p | PKD1P1 |
| ERBIN | hsa-miR-193a-3p | PKD1P1 |
| WAPL | hsa-miR-195-5p | hsa_circ_0000038 |
| SRPRA | hsa-miR-195-5p | hsa_circ_0000038 |
| CALM1 | hsa-miR-195-5p | hsa_circ_0000038 |
| RGPD6 | hsa-miR-195-5p | hsa_circ_0000038 |
| VPS50 | hsa-miR-195-5p | hsa_circ_0000038 |
| SHTN1 | hsa-miR-19a-3p | hsa_circ_0000842 |
| EMSY | hsa-miR-19a-3p | hsa_circ_0000843 |
| CALM1 | hsa-miR-19a-3p | hsa_circ_0000844 |
| FAM234A | hsa-miR-19a-3p | hsa_circ_0000845 |
| ANKRD12 | hsa-miR-19a-3p | hsa_circ_0000846 |
| ZEB2 | hsa-miR-19a-3p | hsa_circ_0000847 |
| PTPA | hsa-miR-19a-3p | hsa_circ_0000848 |
| SHTN1 | hsa-miR-19b-3p | hsa_circ_0000842 |
| EMSY | hsa-miR-19b-3p | hsa_circ_0000842 |
| CALM1 | hsa-miR-19b-3p | hsa_circ_0000842 |
| FAM234A | hsa-miR-19b-3p | hsa_circ_0000842 |
| ANKRD12 | hsa-miR-19b-3p | hsa_circ_0000842 |
| ZEB2 | hsa-miR-19b-3p | hsa_circ_0000842 |
| PTPA | hsa-miR-19b-3p | hsa_circ_0000842 |
| MFSD14A | hsa-miR-200a-3p | hsa_circ_0001648 |
| EMSY | hsa-miR-200a-3p | hsa_circ_0001648 |
| PCNX4 | hsa-miR-200a-3p | hsa_circ_0001648 |
| ZEB2 | hsa-miR-200a-3p | hsa_circ_0001648 |
| PRELID3B | hsa-miR-200a-3p | hsa_circ_0001648 |
| WAPL | hsa-miR-200b-3p | hsa_circ_0001360 |
| SLF2 | hsa-miR-200b-3p | hsa_circ_0001360 |
| ZEB2 | hsa-miR-200b-3p | hsa_circ_0001360 |
| USF3 | hsa-miR-200b-3p | hsa_circ_0001360 |
| WAPL | hsa-miR-200c-3p | hsa_circ_0001360 |
| SLF2 | hsa-miR-200c-3p | hsa_circ_0001360 |
| ZEB2 | hsa-miR-200c-3p | hsa_circ_0001360 |
| USF3 | hsa-miR-200c-3p | hsa_circ_0001360 |
| LCOR | hsa-miR-205-5p | hsa_circ_0001540 |
| PCNX1 | hsa-miR-205-5p | hsa_circ_0001540 |
| ZEB2 | hsa-miR-205-5p | hsa_circ_0001540 |
| MFSD14B | hsa-miR-205-5p | hsa_circ_0001540 |
| MFSD14A | hsa-miR-206 | hsa_circ_0001360 |
| MFSD14A | hsa-miR-206 | RPL9P7 |
| THAP12 | hsa-miR-206 | hsa_circ_0001360 |
| THAP12 | hsa-miR-206 | RPL9P7 |
| ARID2 | hsa-miR-206 | hsa_circ_0001360 |
| ARID2 | hsa-miR-206 | RPL9P7 |
| CALM2 | hsa-miR-206 | hsa_circ_0001360 |
| CALM2 | hsa-miR-206 | RPL9P7 |
| HNRNPA3 | hsa-miR-206 | hsa_circ_0001360 |
| HNRNPA3 | hsa-miR-206 | RPL9P7 |
| GRK3 | hsa-miR-206 | hsa_circ_0001360 |
| GRK3 | hsa-miR-206 | RPL9P7 |
| MFSD14A | hsa-miR-20b-5p | RPL7AP11 |
| KMT5B | hsa-miR-20b-5p | RPL7AP11 |
| EMSY | hsa-miR-20b-5p | RPL7AP11 |
| TAOK3 | hsa-miR-20b-5p | RPL7AP11 |
| UBC | hsa-miR-20b-5p | RPL7AP11 |
| ANKRD12 | hsa-miR-20b-5p | RPL7AP11 |
| PCMTD1 | hsa-miR-20b-5p | RPL7AP11 |
| ARID2 | hsa-miR-212-3p | ZNF322P1 |
| NUP58 | hsa-miR-212-3p | ZNF322P1 |
| LCOR | hsa-miR-21-5p | hsa_circ_0000043 |
| PPP4R3A | hsa-miR-216b-5p | ZNF322P1 |
| VPS41 | hsa-miR-216b-5p | ZNF322P1 |
| WAPL | hsa-miR-217 | hsa_circ_0000160 |
| ARID2 | hsa-miR-217 | hsa_circ_0000161 |
| KMT5A | hsa-miR-217 | hsa_circ_0000162 |
| HNRNPA3 | hsa-miR-217 | hsa_circ_0000163 |
| EMSY | hsa-miR-223-3p | hsa_circ_0000639 |
| FBXO16 | hsa-miR-223-3p | hsa_circ_0000639 |
| MFSD14A | hsa-miR-23a-3p | hsa_circ_0001016 |
| GRK2 | hsa-miR-23a-3p | hsa_circ_0001016 |
| EMSY | hsa-miR-23a-3p | hsa_circ_0001016 |
| KMT5A | hsa-miR-23a-3p | hsa_circ_0001016 |
| NCBP3 | hsa-miR-23a-3p | hsa_circ_0001016 |
| PPP4R3B | hsa-miR-23a-3p | hsa_circ_0001016 |
| ERBIN | hsa-miR-23a-3p | hsa_circ_0001016 |
| VPS50 | hsa-miR-23a-3p | hsa_circ_0001016 |
| MFSD14A | hsa-miR-23b-3p | hsa_circ_0001016 |
| MFSD14A | hsa-miR-23b-3p | ZNF322P1 |
| MFSD14A | hsa-miR-23b-3p | FTH1P20 |
| GRK2 | hsa-miR-23b-3p | hsa_circ_0001016 |
| GRK2 | hsa-miR-23b-3p | ZNF322P1 |
| GRK2 | hsa-miR-23b-3p | FTH1P20 |
| EMSY | hsa-miR-23b-3p | hsa_circ_0001016 |
| EMSY | hsa-miR-23b-3p | ZNF322P1 |
| EMSY | hsa-miR-23b-3p | FTH1P20 |
| KMT5A | hsa-miR-23b-3p | hsa_circ_0001016 |
| KMT5A | hsa-miR-23b-3p | ZNF322P1 |
| KMT5A | hsa-miR-23b-3p | FTH1P20 |
| NCBP3 | hsa-miR-23b-3p | hsa_circ_0001016 |
| NCBP3 | hsa-miR-23b-3p | ZNF322P1 |
| NCBP3 | hsa-miR-23b-3p | FTH1P20 |
| PPP4R3B | hsa-miR-23b-3p | hsa_circ_0001016 |
| PPP4R3B | hsa-miR-23b-3p | ZNF322P1 |
| PPP4R3B | hsa-miR-23b-3p | FTH1P20 |
| ERBIN | hsa-miR-23b-3p | hsa_circ_0001016 |
| ERBIN | hsa-miR-23b-3p | ZNF322P1 |
| ERBIN | hsa-miR-23b-3p | FTH1P20 |
| VPS50 | hsa-miR-23b-3p | hsa_circ_0001016 |
| VPS50 | hsa-miR-23b-3p | ZNF322P1 |
| VPS50 | hsa-miR-23b-3p | FTH1P20 |
| ARID2 | hsa-miR-24-3p | EEF1A1P11 |
| ARID2 | hsa-miR-24-3p | PKD1P1 |
| ARID2 | hsa-miR-24-3p | EEF1A1P9 |
| USF3 | hsa-miR-24-3p | EEF1A1P11 |
| USF3 | hsa-miR-24-3p | PKD1P1 |
| USF3 | hsa-miR-24-3p | EEF1A1P9 |
| RUBCN | hsa-miR-24-3p | EEF1A1P11 |
| RUBCN | hsa-miR-24-3p | PKD1P1 |
| RUBCN | hsa-miR-24-3p | EEF1A1P9 |
| LCOR | hsa-miR-27a-3p | ZNF322P1 |
| LCOR | hsa-miR-27a-3p | FTH1P20 |
| LCOR | hsa-miR-27a-3p | EEF1A1P9 |
| KMT5B | hsa-miR-27a-3p | ZNF322P1 |
| KMT5B | hsa-miR-27a-3p | FTH1P20 |
| KMT5B | hsa-miR-27a-3p | EEF1A1P9 |
| ARID2 | hsa-miR-27a-3p | ZNF322P1 |
| ARID2 | hsa-miR-27a-3p | FTH1P20 |
| ARID2 | hsa-miR-27a-3p | EEF1A1P9 |
| PCNX1 | hsa-miR-27a-3p | ZNF322P1 |
| PCNX1 | hsa-miR-27a-3p | FTH1P20 |
| PCNX1 | hsa-miR-27a-3p | EEF1A1P9 |
| XPO1 | hsa-miR-27a-3p | ZNF322P1 |
| XPO1 | hsa-miR-27a-3p | FTH1P20 |
| XPO1 | hsa-miR-27a-3p | EEF1A1P9 |
| RGPD6 | hsa-miR-27a-3p | ZNF322P1 |
| RGPD6 | hsa-miR-27a-3p | FTH1P20 |
| RGPD6 | hsa-miR-27a-3p | EEF1A1P9 |
| RGPD5 | hsa-miR-27a-3p | ZNF322P1 |
| RGPD5 | hsa-miR-27a-3p | FTH1P20 |
| RGPD5 | hsa-miR-27a-3p | EEF1A1P9 |
| USF3 | hsa-miR-27a-3p | ZNF322P1 |
| USF3 | hsa-miR-27a-3p | FTH1P20 |
| USF3 | hsa-miR-27a-3p | EEF1A1P9 |
| KMT5B | hsa-miR-29a-3p | hsa_circ_0001020 |
| EMSY | hsa-miR-29a-3p | hsa_circ_0001021 |
| IFI30 | hsa-miR-29a-3p | hsa_circ_0001022 |
| PPP4R3B | hsa-miR-29a-3p | hsa_circ_0001023 |
| USF3 | hsa-miR-29a-3p | hsa_circ_0001024 |
| KMT5B | hsa-miR-29b-3p | hsa_circ_0001020 |
| EMSY | hsa-miR-29b-3p | hsa_circ_0001021 |
| IFI30 | hsa-miR-29b-3p | hsa_circ_0001022 |
| PPP4R3B | hsa-miR-29b-3p | hsa_circ_0001023 |
| USF3 | hsa-miR-29b-3p | hsa_circ_0001024 |
| KMT5B | hsa-miR-29c-3p | hsa_circ_0001020 |
| EMSY | hsa-miR-29c-3p | hsa_circ_0001021 |
| IFI30 | hsa-miR-29c-3p | hsa_circ_0001022 |
| PPP4R3B | hsa-miR-29c-3p | hsa_circ_0001023 |
| USF3 | hsa-miR-29c-3p | hsa_circ_0001024 |
| MAN1A2 | hsa-miR-30c-5p | hsa_circ_0000091 |
| SLF2 | hsa-miR-30c-5p | hsa_circ_0000091 |
| THAP12 | hsa-miR-30c-5p | hsa_circ_0000091 |
| XPO1 | hsa-miR-30c-5p | hsa_circ_0000091 |
| HNRNPA3 | hsa-miR-30c-5p | hsa_circ_0000091 |
| SRPRA | hsa-miR-326 | hsa_circ_0000639 |
| ERBIN | hsa-miR-326 | hsa_circ_0000639 |
| SRPRA | hsa-miR-330-5p | hsa_circ_0000639 |
| ERBIN | hsa-miR-330-5p | hsa_circ_0000639 |
| WAPL | hsa-miR-338-3p | TPM3P8 |
| WAPL | hsa-miR-338-3p | RPL21P119 |
| WAPL | hsa-miR-338-3p | EEF1A1P11 |
| WAPL | hsa-miR-338-3p | PKD1P1 |
| WAPL | hsa-miR-338-3p | EEF1A1P9 |
| WAPL | hsa-miR-338-3p | HMGN2P41 |
| PCNX1 | hsa-miR-33a-5p | hsa_circ_0000160 |
| ERBIN | hsa-miR-33a-5p | hsa_circ_0000160 |
| PCNX1 | hsa-miR-33b-5p | hsa_circ_0000160 |
| ERBIN | hsa-miR-33b-5p | hsa_circ_0000160 |
| MFSD14A | hsa-miR-340-5p | hsa_circ_0000019 |
| LCOR | hsa-miR-340-5p | hsa_circ_0000019 |
| KMT5A | hsa-miR-340-5p | hsa_circ_0000019 |
| RGPD6 | hsa-miR-340-5p | hsa_circ_0000019 |
| SRPRA | hsa-miR-34a-5p | hsa_circ_0000038 |
| HSPA1B | hsa-miR-34a-5p | hsa_circ_0000038 |
| SRPRA | hsa-miR-34c-5p | hsa_circ_0000038 |
| HSPA1B | hsa-miR-34c-5p | hsa_circ_0000038 |
| KIF1BP | hsa-miR-363-3p | RPL9P7 |
| LCOR | hsa-miR-363-3p | RPL9P7 |
| KMT5B | hsa-miR-363-3p | RPL9P7 |
| SRPRA | hsa-miR-363-3p | RPL9P7 |
| ZEB2 | hsa-miR-363-3p | RPL9P7 |
| MORC3 | hsa-miR-363-3p | RPL9P7 |
| ERBIN | hsa-miR-363-3p | RPL9P7 |
| PCMTD1 | hsa-miR-363-3p | RPL9P7 |
| RGS3 | hsa-miR-363-3p | RPL9P7 |
| KMT5A | hsa-miR-382-5p | hsa_circ_0001648 |
| KMT5A | hsa-miR-382-5p | hsa_circ_0000842 |
| MFSD14B | hsa-miR-382-5p | hsa_circ_0001648 |
| MFSD14B | hsa-miR-382-5p | hsa_circ_0000842 |
| EMSY | hsa-miR-421 | hsa_circ_0000160 |
| TOMM70 | hsa-miR-421 | hsa_circ_0000160 |
| WAPL | hsa-miR-424-5p | hsa_circ_0000038 |
| SRPRA | hsa-miR-424-5p | hsa_circ_0000038 |
| CALM1 | hsa-miR-424-5p | hsa_circ_0000038 |
| RGPD6 | hsa-miR-424-5p | hsa_circ_0000038 |
| VPS50 | hsa-miR-424-5p | hsa_circ_0000038 |
| LCOR | hsa-miR-425-5p | EEF1A1P11 |
| LCOR | hsa-miR-425-5p | EEF1A1P9 |
| PPP4R3A | hsa-miR-425-5p | EEF1A1P11 |
| PPP4R3A | hsa-miR-425-5p | EEF1A1P9 |
| WAPL | hsa-miR-429 | hsa_circ_0001360 |
| SLF2 | hsa-miR-429 | hsa_circ_0001360 |
| ZEB2 | hsa-miR-429 | hsa_circ_0001360 |
| USF3 | hsa-miR-429 | hsa_circ_0001360 |
| SRPRA | hsa-miR-449a | hsa_circ_0000038 |
| HSPA1B | hsa-miR-449a | hsa_circ_0000038 |
| SRPRA | hsa-miR-449b-5p | hsa_circ_0000038 |
| HSPA1B | hsa-miR-449b-5p | hsa_circ_0000038 |
| SHTN1 | hsa-miR-494-3p | hsa_circ_0000842 |
| EMSY | hsa-miR-494-3p | hsa_circ_0000842 |
| HNRNPA3 | hsa-miR-494-3p | hsa_circ_0000842 |
| WAPL | hsa-miR-497-5p | hsa_circ_0000038 |
| SRPRA | hsa-miR-497-5p | hsa_circ_0000038 |
| CALM1 | hsa-miR-497-5p | hsa_circ_0000038 |
| RGPD6 | hsa-miR-497-5p | hsa_circ_0000038 |
| VPS50 | hsa-miR-497-5p | hsa_circ_0000038 |
| VPS41 | hsa-miR-505-3p | hsa_circ_0000019 |
| VPS41 | hsa-miR-505-3p | hsa_circ_0001540 |
| EMSY | hsa-miR-543 | hsa_circ_0000311 |
| EMSY | hsa-miR-543 | hsa_circ_0000842 |
| LCOR | hsa-miR-590-5p | hsa_circ_0000043 |
| LCOR | hsa-miR-590-5p | EEF1A1P11 |
| MFSD14A | hsa-miR-613 | hsa_circ_0001360 |
| THAP12 | hsa-miR-613 | hsa_circ_0001360 |
| ARID2 | hsa-miR-613 | hsa_circ_0001360 |
| CALM2 | hsa-miR-613 | hsa_circ_0001360 |
| HNRNPA3 | hsa-miR-613 | hsa_circ_0001360 |
| GRK3 | hsa-miR-613 | hsa_circ_0001360 |
| MFSD14A | hsa-miR-613 | RPL9P7 |
| THAP12 | hsa-miR-613 | RPL9P7 |
| ARID2 | hsa-miR-613 | RPL9P7 |
| CALM2 | hsa-miR-613 | RPL9P7 |
| HNRNPA3 | hsa-miR-613 | RPL9P7 |
| GRK3 | hsa-miR-613 | RPL9P7 |
| EMSY | hsa-miR-653-5p | hsa_circ_0001684 |
| WAPL | hsa-miR-7-5p | hsa_circ_0000160 |
| ARID2 | hsa-miR-7-5p | hsa_circ_0000160 |
| KMT5A | hsa-miR-7-5p | hsa_circ_0000160 |
| ANKRD12 | hsa-miR-7-5p | hsa_circ_0000160 |
| SLF2 | hsa-miR-98-5p | hsa_circ_0000038 |
| INTS6L | hsa-miR-98-5p | hsa_circ_0000160 |

**Figure S1:** Venn Diagram of predicted microRNAs targeting DEMs, DELs and DECs. Abbreviations: DEMs, differentially expressed mRNAs; DELs, differentially expressed lncRNAs; DECs, differentially expressed circRNAs.


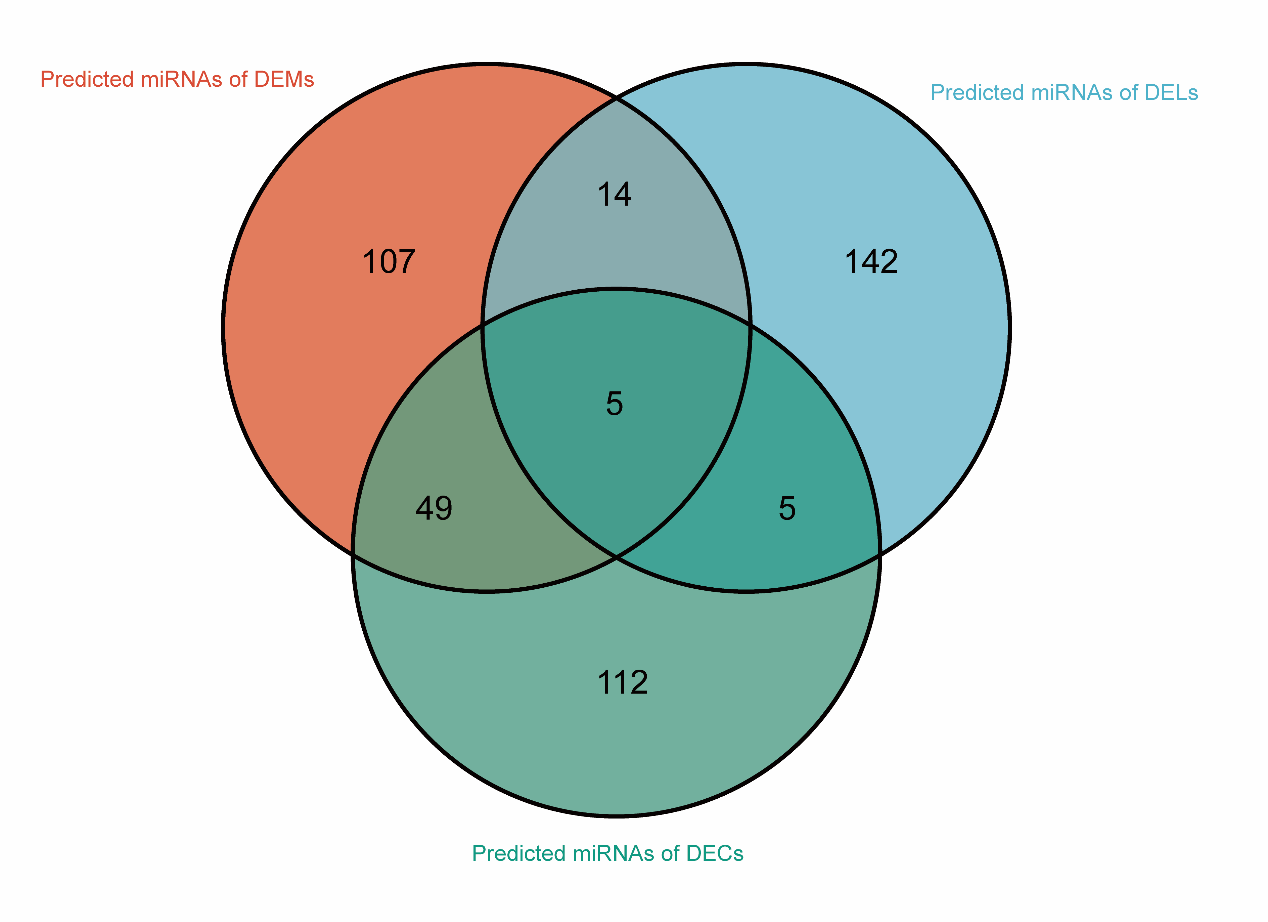

Supplement: Supplementary Materials — Table S1: all upregulated and downregulated DEMs between the normal controls and the CHD samples. Table S2: all upregulated and downregulated DELs between the normal controls and the CHD samples. Table S3: all upregulated and downregulated DECs between the normal controls and the CHD samples. Table S4: enrichment analyses of differentially expressed mRNAs. Table S5: enrichment analyses of module hub genes. Table S6: complete list of competing endogenous RNA network pairs. Figure S1: Venn diagram of predicted microRNAs targeting DEMs, DELs, and DECs. Abbreviations: DEMs, differentially expressed mRNAs; DELs, differentially expressed lncRNAs; and DECs, differentially expressed circRNAs. [file 6682183.f1.zip › 6682183.f1/Supplementary File.docx]
