# Supplementary material for: Identifying a Serum Exosomal-Associated lncRNA/circRNA-miRNA-mRNA Network in Coronary Heart Disease
Source: Cardiol Res Pract. 2021 Jun 23;2021:6682183. doi: 10.1155/2021/6682183 (PMC8249161; doi:10.1155/2021/6682183)
Supplement: Supplementary Materials — Table S1: all upregulated and downregulated DEMs between the normal controls and the CHD samples. Table S2: all upregulated and downregulated DELs between the normal controls and the CHD samples. Table S3: all upregulated and downregulated DECs between the normal controls and the CHD samples. Table S4: enrichment analyses of differentially expressed mRNAs. Table S5: enrichment analyses of module hub genes. Table S6: complete list of competing endogenous RNA network pairs. Figure S1: Venn diagram of predicted microRNAs targeting DEMs, DELs, and DECs. Abbreviations: DEMs, differentially expressed mRNAs; DELs, differentially expressed lncRNAs; and DECs, differentially expressed circRNAs. [file 6682183.f1.zip › 6682183.f1/SupplementaryFigure S1.docx]

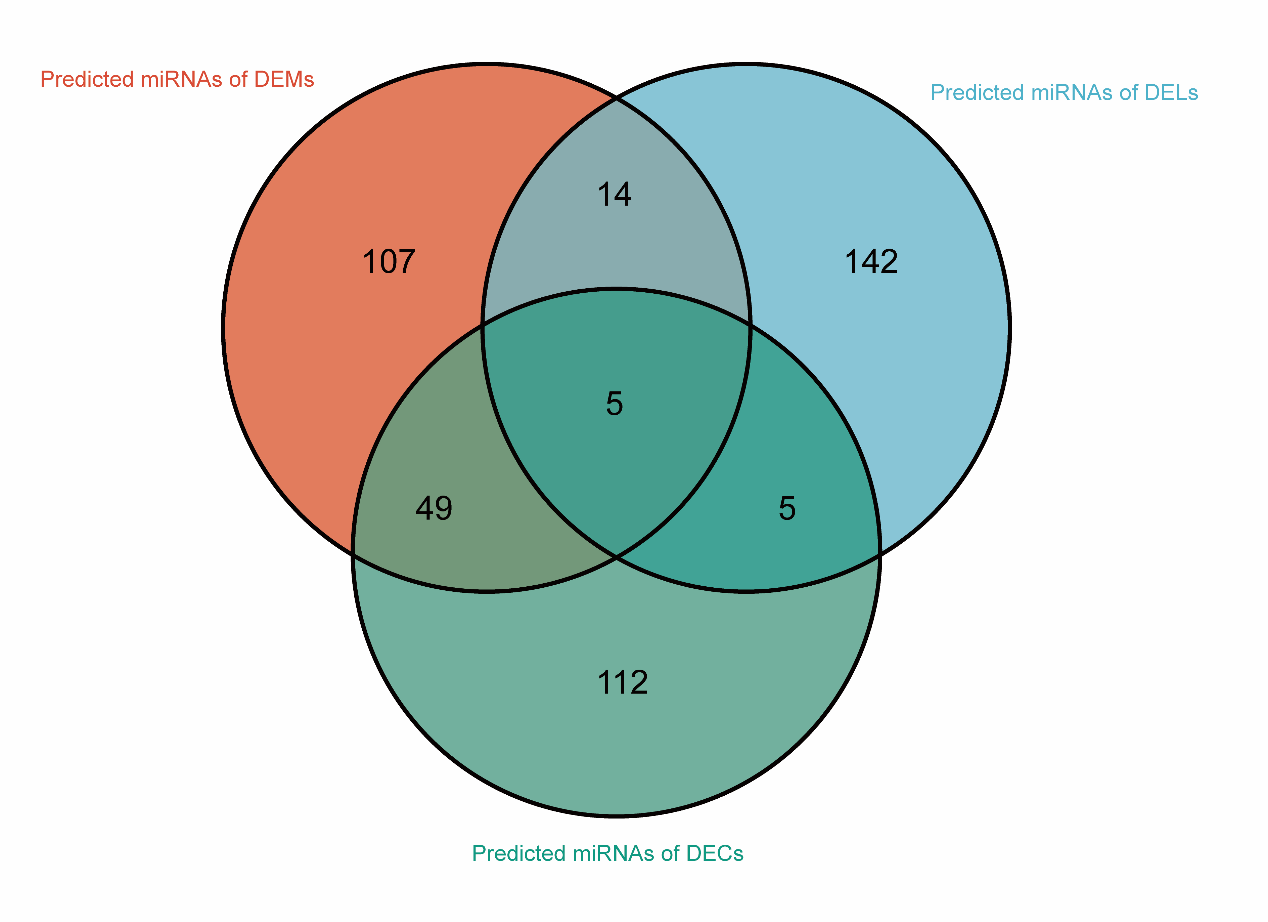


**SupplementaryFigure S1:** Venn Diagram of predicted microRNAs targeting DEMs, DELs and DECs. Abbreviations: DEMs, differentially expressed mRNAs; DELs, differentially expressed lncRNAs; DECs, differentially expressed circRNAs.
